# On joy and sorrow: Neuroimaging meta-analyses of music-induced emotion

**DOI:** 10.1162/imag_a_00425

**Published:** 2025-01-16

**Authors:** Nieves Fuentes-Sánchez, Alejandro Espino-Payá, Sabine Prantner, Dean Sabatinelli, M. Carmen Pastor, Markus Junghöfer

**Affiliations:** Departamento de Psicología, Universidad de Castilla-La Mancha, Facultad de Medicina, Albacete, Spain; Institute for Biomagnetism and Biosignalanalysis, University of Münster, Münster, Germany; Otto Creutzfeldt Center for Cognitive and Behavioral Neuroscience, University of Münster, Münster, Germany; Departamento de Psicología Básica, Clínica y Psicobiología, Universitat Jaume I, Facultad de Ciencias de la Salud, Castelló de la Plana, Spain; Institute of Psychology, Clinical Psychology and Psychotherapy in Childhood and Adolescence, University of Osnabrueck, Osnabrueck, Germany; Department of Psychology, University of Georgia, Athens, GA, United States

**Keywords:** emotion, music, laterality, fMRI, ALE meta-analysis

## Abstract

Prior neuroimaging studies of music-evoked emotions have shown that music listening involves the activation of both cortical and subcortical regions. However, these regions could be differentially activated by music stimuli with varying affective valence and arousal. To better understand the neural correlates involved in the processing of pleasant and unpleasant emotions induced by music, while also considering the effect of arousal, we conducted a quantitative activation likelihood estimate (ALE) meta-analysis. We performed separate ALE analyses for the overall brain activation evoked by listening to emotional music (40 studies), for the brain activation during listening to unpleasant music (15 studies), for the brain activation while listening to pleasant music (17 studies), and for the brain activation while listening to emotional contrasted with neutral music (8 studies). Our results revealed the activation of a range of cortical and subcortical regions, including the amygdala, insula, striatum, thalamus, hippocampus, anterior cingulate gyrus, and superior temporal gyrus. Moreover, our findings indicated that certain regions were specifically activated based on the hedonic valence and arousal of the stimuli. Particularly, whereas the anterior cingulate cortex (ACC), dorsal striatum, and thalamus were dependent on arousal effects, amygdala activation was dependent on hedonic valence. The identification of brain networks preferentially activated during listening to pleasant and unpleasant music provides valuable clinical insights for the development of therapies targeting psychological disorders associated with emotion reactivity problems.

## 1 Introduction

Music has been present in different cultures since ancient times. Listening to music, however, does not seem to be a survival-relevant activity, which suggests that other factors might explain the origin or evolution of music (Zatorre & Salimpoor, 2013). In this regard, several theories attempt to explain the evolution of music, such as the explanation that posits that social bonding is a key factor in the biological and cultural evolution of music (Savage et al., 2021), or the theory that argues that music evolved as a signal for cooperation ability and infant care (Mehr et al., 2021). In relation to this research, another possible factor that could explain the evolution of music, potentially interacting with the aforementioned core factors (Mehr et al., 2021), is the capacity of music to convey, induce, and regulate emotions (Hauser & McDermott, 2003). Prior research has demonstrated that music can evoke powerful emotions, measurable through experiential, peripheral–physiological, and brain reactivity levels (Blood & Zatorre, 2001;Fuentes-Sánchez et al., 2021b). This makes music a valuable stimulus for investigating emotional processing (Koelsch, 2014). To this respect, music serves as a complementary stimulus to other affective stimuli commonly used in emotion research, such as affective scenes, facial expressions, or movies. Music has also garnered significant interest from researchers in recent years due to its high ecological validity. Consequently, there has been a surge in scientific studies using music to influence behavior (Koelsch, 2020).

Previous neuroimaging meta-analyses of music processing, such as byKoelsch (2014,^2020^), have demonstrated the existence of a large network of neural correlates of emotions evoked by music. These analyses have shown that music activates not only emotion-related areas such as the amygdala, insula, or striatum, but also the auditory cortex, hippocampus, and secondary somatosensory cortex. This suggests that these areas typically associated with cognitive and perceptual processes also have a significant role in music processing (Koelsch, 2020). However, in prior meta-analyses, the potential impact of valence (pleasant vs. unpleasant), arousal, or specific emotions induced has been scarcely investigated. Certainly, music-evoked emotion is quite powerful, but it may be that subtypes of emotional music may be associated with distinct patterns of brain reactivity. In this regard, to our knowledge, only one meta-analysis (Mas-Herrero et al., 2021) has investigated the effect of hedonic valence on brain activations during music listening. However, this work primarily focused on brain activations during listening to pleasant music, without exploring other emotional categories, such as the processing of unpleasant music. Therefore, it would be interesting to conduct further meta-analyses that investigate not only brain responses during listening to pleasant music, but also to unpleasant music. Additionally, examining brain activations in relation to the emotional intensity of the music would provide deeper insights into the neural mechanisms of music-evoked emotions.

Discrete and dimensional models have increasingly been used in the field of music and emotions (Eerola & Vuoskoski, 2011;Fuentes-Sánchez et al., 2021a;Song et al., 2016). The discrete emotion model argues for the existence of a limited number of basic emotions, which have specific and distinguishing neurophysiological and behavioral patterns (Ekman, 1992). Findings obtained from this approach have shown that, generally, each discrete emotion is associated with the activation of some specific areas in the brain. For example, disgust typically evokes insula activation while fear predominantly activates the amygdala (Murphy et al., 2003;Vytal & Hamann, 2010). By contrast, the dimensional approach considers that all emotions underlie more general dimensions such as valence/arousal, positive/negative activation, or approach/withdrawal (Bradley, 2000;Bradley et al., 2001;Barrett & Wager, 2006;Lang et al., 1997). Among the different dimensional models of emotion (Rubin & Talarico, 2009), the bidimensional model proposed by Peter Lang is one of the most widely accepted in the field (Lang & Bradley, 2013). This model posits that emotions arise from the activation of two opposite motivational systems in the brain: a defensive system associated with unpleasant affect and an appetitive system liked to pleasant affect, with both systems varying in the intensity of their activation (Bradley, 2000;Bradley et al., 2001;Barrett & Wager, 2006;Lang et al., 1997). In this framework, factor analyses of emotional language (Bradley & Lang, 1994;Osgood et al., 1957) identified two main factors underlying the motive-circuit brain model: hedonic valence (positive/pleasant/appetitive vs. negative/aversive/defensive) and emotional arousal (intensity of activation) (Lang & Bradley, 2013). From this perspective, findings have revealed that many brain areas are activated by multiple emotions (e.g.,Lindquist et al., 2012). For instance, the amygdala is not only activated by fear-inducing stimuli, as suggested by the discrete model of emotions, but also by emotionally significant stimuli in general, including those with positive hedonic valence (Barrett & Wager, 2006;Sabatinelli et al., 2005;Zald, 2003).

Most neuroimaging studies employing music stimuli do not assess emotion states using both discrete and dimensional measures, making it difficult to extract potentially distinct brain areas (Hamann, 2012). For example, within the discrete approach model, studies have mainly focused on the contrast between happiness and fear (Koelsch & Skouras, 2014;Koelsch et al., 2021) or happiness and sadness (Brattico et al., 2011). Likewise, within the dimensional model, some work has focused on pleasant versus unpleasant emotion (Koelsch et al., 2006), while other studies have focused on the relationship between consonance and dissonance (Suzuki et al., 2008), or examined the relationship between some regional brain activity and the intensity of chills evoked by different pieces of music (Blood & Zatorre, 2001).

No meta-analysis has been conducted thus far that investigates the neural correlates of music-induced emotions considering both the affective valence and arousal of music. Given that the functional neuroanatomy of emotional states has been of great interest within the field of cognitive neuroscience and, considering the existence of multiple meta-analyses that have focused on other modes of emotional evocation (Murphy et al., 2003;Wager et al., 2003), the justification to carry out a valence/arousal-focused meta-analysis is clear. The current meta-analyses include three different aims. Firstly, one meta-analysis aims to replicate and extend prior meta-analyses of brain activity during listening to emotional music, in which contrasts with non-musical condition were not included to ensure that the effects found in the auditory cortex are not due to the contrast of music versus no music (Koelsch, 2014,^2020^). Next, we investigated the brain structures involved in pleasant relative to unpleasant emotions evoked by music (i.e., the hedonic valence effect). For this purpose, direct ALE meta-analyses were conducted between pleasant and unpleasant music (unpleasant contrasted to pleasant music to test the effect of unpleasant music; and pleasant contrasted to unpleasant music to test the effect of pleasant music). Here we consider*pleasant*and*unpleasant*emotions as general labels that include both discrete and dimensional classifications of emotion states. For example, within the label of pleasant emotions, we included studies that used music to induce states of joy, happiness, pleasantness, liking, consonance, etc., whereas studies that used music to induce unpleasant emotion states included studies inducing fear, unpleasantness, dislike, dissonance, etc. Within the unpleasant category, we chose not to include studies that employed what is commonly described as sad music, because it is often rated as neutral or even pleasant for the listeners. For example, recent literature showed that if the dimensional model is used to evaluate sad music, it is rated as neutral (Fuentes-Sánchez et al., 2021a,2021b). Lastly, in order to test the effect of the arousal independent from valence, the brain activity during the listening of emotional music (pleasant and unpleasant music) was contrasted to the neural activity during listening of neutral music. The arousal contrast is expected to be consistent with prior meta-analyses of music-induced emotion (Koelsch, 2014,^2020^). If pleasant and unpleasant music evokes a unique emotional experience, the valence-specific contrasts may reveal distinct patterns of regional brain activity.

## 2 Methods

### 2.1 Search method and study selection

The literature search was conducted through the following databases: PubMed (www.ncbi.nlm.nih.gov/pubmed), Scopus (Elsevier, Amsterdam, Netherlands), and Web of Science (https://www.webofscience.com). Additionally, citations and reference lists from relevant articles were reviewed. Eligible studies were experimental studies that investigated brain responses during the listening of music using fMRI (functional magnetic resonance imaging) or PET (positron emission tomography). The terms used to conduct the search were: [(“Emotion” OR “Affect” OR “Mood”) AND (“Music” OR “Excerpts” OR “Song”) AND (“fMRI” OR “Functional Magnetic Resonance Imaging” OR “PET” OR “Positron Emission Tomography”)].

As shown inFigure 1, studies that investigated other psychological processes associated with explicit tasks during music listening (e.g., emotion regulation, memory, etc.) were not included (n = 23). To circumvent complex interactions of language- and music-induced emotions, studies that used music with lyrics were also excluded (n = 10). Additionally, to be included in the meta-analysis, studies had to target adult participants (≥18 years) and non-clinical samples^†^. Also, reviews, meta-analyses, dissertations, and conference abstracts were excluded. Lastly, taking into account the linguistic capacities of the authors, only studies published in English, Spanish, or German languages were included, with no restrictions based on the year of publication (cutoff date: August 23, 2022).

**Fig. 1.**
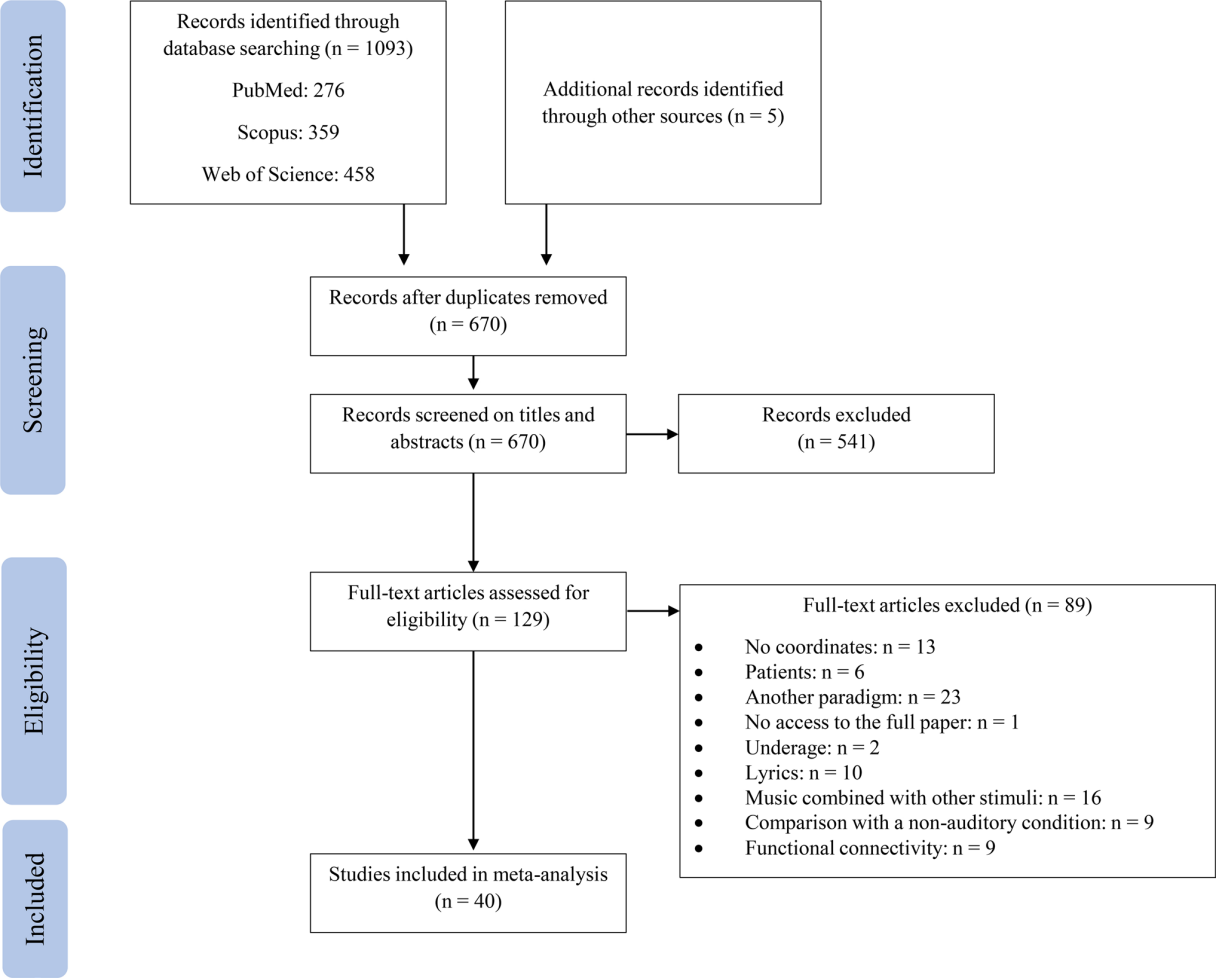
Flow diagram of article selection following PRISMA guidelines.

For the ALE meta-analysis^‡^, the final inclusion criteria were that eligible studies should include whole brain analyses and not just specific regions of interest and should contain a complete list of stereotaxic coordinates (i.e., Montreal Neurological Institute [MNI] or Talairach space) (Talairach & Tournoux, 1988). Additionally, contrasts that compared music listening with a non-auditory condition (e.g., rest) were not included (n = 9). Likewise, as the aim of the current meta-analysis is to investigate brain activations, functional connectivity results were not considered (n = 9). In eligible studies where this information was missing, the authors were contacted.

The search generated a total of 1093 potential studies. Five additional studies were obtained from other relevant articles. Therefore, 1098 studies were identified. After excluding duplicates (n = 428), a total of 670 studies were screened by 2 independent researchers (N.F.-S. and S.P.), based on titles and abstracts. In this step, the researchers read the title of the paper and the abstract and discarded those studies that did not aim to investigate brain activations during music listening. As a result, of the 670 studies, 129 studies were assessed for eligibility. After full article inspection, 40 studies were used for the final ALE meta-analysis (seeFig. 1).

The meta-analysis was performed following the methodological guidelines byV. I. Mueller et al. (2018)(seeFig. 2).

**Fig. 2.**
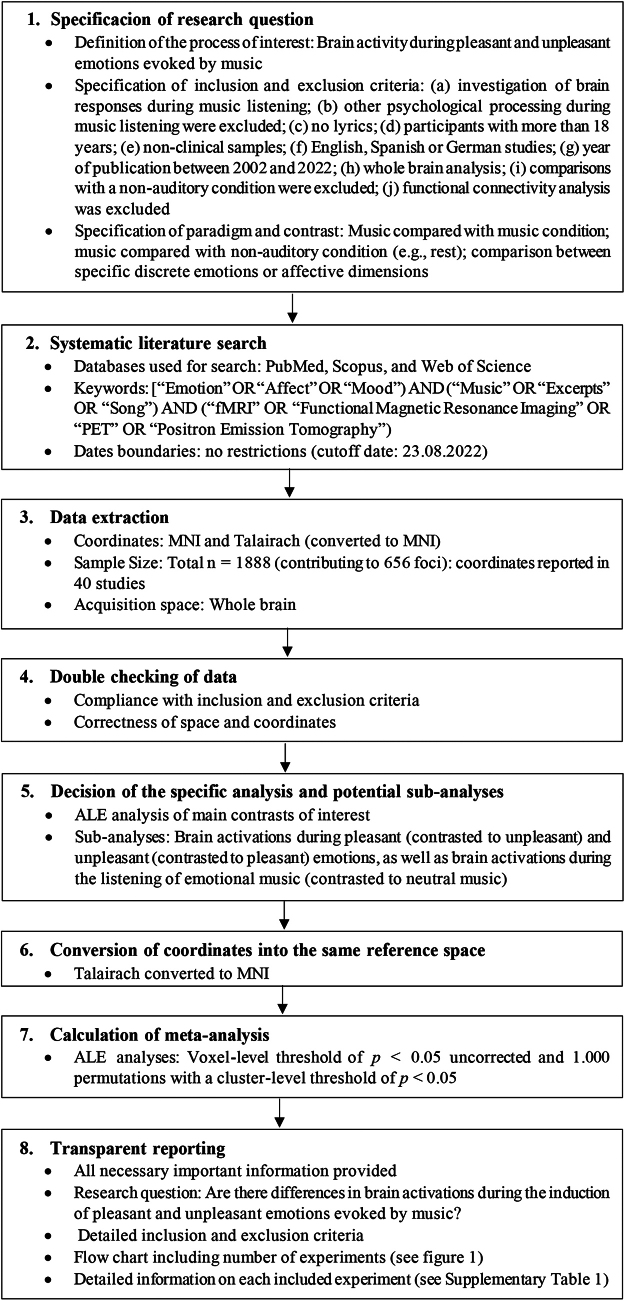
Flowchart illustrating all important steps of the meta-analysis following the guidelines byV. I. Mueller et al. (2018).

### 2.2 Data analysis

Relevant information (reference space, sample size, coordinates of activation, type of music, duration of music, etc.) was obtained by three researchers (N.F.-S., A.E.-P., & S.P.) from the selected articles (N = 40) for the posterior analysis. All information was double-checked by a different researcher.

To identify consistent brain activation across studies, the activation likelihood estimation (ALE) approach (Eickhoff et al., 2009,^2012^,^2017^;Laird et al., 2005;Turkeltaub et al., 2012) was carried out. Talairach coordinates (Talairach & Tournoux, 1988) were converted to MNI using GingerALE, and all results were presented in MNI space. After preparing the selected contrasts, the ALE analysis was performed using GingerALE 3.0.2 software (http://brainmap.org/ale) (Lancaster et al., 2007). For the general analysis and sub-analyses, the family-wise error (FWE) method was used to correct for multiple comparisons using a voxel-level threshold of*p*< 0.05 uncorrected and 1000 permutations with a cluster-level threshold of*p*< 0.05. The resulting peak coordinates are reported in MNI. To visualize the meta-analysis results, the resulting output was overlaid onto anatomical axial, coronal, and sagittal slice images in MNI space.

## 3 Results

### 3.1 Included articles

The final meta-analysis included 40 studies, including 1888 subjects and 656 foci from 97 different contrasts (seeTable 1). A complete list of studies and their characteristics can be seen inSupplementary Table 1. In accordance with the three objectives of this study, four separate sub-analyses were conducted. Firstly, in order to replicate prior meta-analyses, a general analysis was performed investigating brain activations during listening to music. Secondly, with the aim to test the effect of hedonic valence, two separate sub-analyses were conducted investigating specific brain activations evoked by listening to unpleasant (in contrast to pleasant) and to pleasant (in contrast to unpleasant) music excerpts. Lastly, to test the effect of arousal, a meta-analysis in which the brain activations evoked by listening to emotional music (pleasant and unpleasant) in contrast to neutral music was performed. In the former, 15 studies were included in an ALE analysis to investigate brain activity during listening to unpleasant (minus pleasant) music (with 284 subjects and 97 foci from 17 different contrasts). In the second, 17 studies were included in an ALE analysis (with 421 subjects and 174 foci from 22 different contrasts) to investigate brain activity evoked by pleasant (minus unpleasant) music. In the final ALE analysis, 8 studies were included (with 272 subjects and 89 foci from 12 different contrasts). The studies included in each ALE analysis are listed inSupplementary Table 4.

**Table 1.** Contrasts included in the general meta-analysis.

Study	Modality	Nr. of subjects	Contrast	Nr. of foci
Aubé et al. (2015)	fMRI	47	Fear > neutral	2
			Happiness > neutral	2
			Music correlations with intensity	2
Altenmüller et al. (2014)	fMRI	18	Positive pieces > less positive pieces	9
Berthold-Losleben et al. (2018)	fMRI	32 (16 females)	Positive > neutral	12
Brattico et al. (2016)	fMRI	29 (15 females)	Like > dislike	17
			Dislike > like	4
			Happy > sad	4
			Sad > happy	1
Blood et al. (1999)	PET	10 (5 females)	Positive correlations with dissonance	3
			Negative correlations with dissonance	5
			Positive correlations with ratings of pleasantness	2
			Negative correlations with ratings of pleasantness	2
Blood and Zatorre (2001)	fMRI	10 (5 females)	Positive correlations with chill intensity	10
			Negative correlations with chills intensity	7
			Increases in rCBF for subject-selected music minus control	10
			Decreases in rCBF for subject-selected music minus control	7
			Positive correlations with pleasantness	9
			Negative correlation with pleasantness	6
			Positive correlation with emotional intensity	8
			Negative correlation with emotional intensity	7
Chapin et al. (2010)	fMRI	14 (9 females)	Emotional arousal	2
			Emotional arousal: Experienced vs. inexperienced	9
Daly et al. (2019)	fMRI	21	Bold co-variation with reported felt affect (but not with movement) during generated music listening task	19
			Bold co-variation with reported felt affect (but not with movement) during classical music listening task	12
Flores-Gutiérrez et al. (2007)	fMRI	6	Pleasant	11
			Unpleasant	21
			Excitement	8
			Calmness	8
Jeong et al. (2011)	fMRI	15	Happy music > sad music	2
Koelsch et al. (2021)	fMRI	24 (13 females)	Joy > fear	28
Koelsch et al. (2013)	fMRI	18	Joy > fear	4
			Fear > joy	1
Koelsch et al. (2018)	fMRI	24	GLM results of the comparison between emotion conditions. Joy > fear	2
			GLM results of the comparison between emotion conditions. Fear > joy	1
			GLM results of the comparison between emotion conditions. Fear > neutral	4
			GLM results of the comparison between emotion conditions. Neutral > fear	2
			GLM results of the comparison between emotion conditions. Joy > neutral	3
Koelsch et al. (2008)	fMRI	20 (10 females)	Irregular > regular chords	4
Koelsch et al. (2006)	fMRI	11 (5 females)	Unpleasant > pleasant	6
			Pleasant > unpleasant	6
Khalfa et al. (2005)	fMRI	13 (5 females)	Minor > major	3
			Mode > tempo interaction	3
Kim et al. (2017)	fMRI	23 (13 females)	Common effect in BOLD for individual differences in dislike of dissonant	7
			Inter-subject correlation in BOLD and rating contrast	1
Kleipzig et al. (2020)	fMRI	16 (12 females)	Music with individual pleasant chill > music without chill	10
Kornysheva et al. (2010)	fMRI	18	Beautiful vs. not beautiful rhythms	10
Lehne et al. (2014)	fMRI	25	Positive correlation with tension	1
			Tension (versions with dynamics) > tension (version without dynamics)	2
			Tension increase > tension decrease	7
Lepping et al. (2016)	fMRI	20	Positive > negative	4
			Negative > positive	2
Liu et al. (2018)	fMRI	48 (25 females)	Fast–slow	2
			Medium–slow	6
Martínez-Molina et al. (2016)	fMRI	45	Pleasure ratings as a parametric effect	1
Matthews et al. (2020)	fMRI	54	Medium > high rhythmic complexity contrast	36
Menon and Levitin (2005)	fMRI	13 (7 females)	Scrambled music > pleasant music	6
Mizuno and Sugishita (2007)	fMRI	18	Major–neutral	8
			Minor–neutral	5
			Major–minor	6
			Minor–major	3
Mitterschiffhaler et al. (2007)	fMRI	16 (10 females)	Happy > neutral	11
			Sad > neutral	7
			Sad > neutral	2
K. Mueller et al. (2011)	fMRI	20 (7 females)	Joyful instrumental tunes > reversed dissonant	7
K. Mueller et al. (2015)	fMRI	23 (13 females)	Correlation with pleasantness	16
Oetken et al. (2017)	fMRI	20 (11 females)	Musically induced mood (happy vs. sad vs. neutral) X self-evaluation vs. lexical decision making	4
Okuya et al. (2017)	fMRI	20 (2 females)	Happy	7
			Sad	1
			Fear	19
Park et al. (2013)	fMRI	12	Happiness vs. control	19
			Fear vs. control	17
Salimpoor et al. (2013)	fMRI	19 (10 females)	Music purchased vs. not purchased	8
			Music purchased vs. not purchased participants highly rewarding	5
Salimpoor et al. (2011)	PET and fMRI	10	PET pleasant–neutral	2
			fMRI pleasant > neutral	7
Shany et al. (2019)	fMRI	40 (for fMRI 31 in Ligeti; 28 in Glass, and 28 in Mussorgsky)	Brain activation as a function of surprise level	32
Sievers et al. (2021)	fMRI	20 (11 females)	Music listening vs. neutral	2
Skouras et al. (2014)	fMRI	32	Joy > fear (3 tesla)	4
			Joy > fear (1.5 tesla)	4
			Neutral > sad & happy	2
Suzuki et al. (2008)	PET	13	Beautiful consonance vs. ugly dissonance	1
			Ugly dissonance vs. beautiful consonance	3
			Minor vs. major	1
			Beautiful major vs. ugly major	2
			Beautiful minor vs. ugly minor	7
			Beautiful major vs. beautiful minor	1
			Beautiful minor vs. beautiful major	2
Tabei (2015)	fMRI	17 (10 females)	Felt > passive listening	3
Trost et al. (2012)	fMRI	16 (9 females)	Correlated with tension	8
			Correlated with joy	4
			Correlated with peacefulness	9
			Correlated with sadness	3
			Correlated with high arousal	13
			Correlated with low arousal	3
			Correlated with positive valence	11
			Correlated with negative valence	3

Note: In those cases where the number of women was not included in the article, the number is not included in this table either.

Note: Experimental literature has demonstrated that musical tempo and mode are important factors in the induction of emotions in listeners. Specifically, music with fast tempo typically evokes pleasant emotions such as happiness, whereas music with slow tempo preferentially induces sadness (Liu et al., 2018). Likewise, major mode music is capable of inducing pleasant emotions, whereas minor mode is typically associated with unpleasant emotions (Bai et al., 2016;Juslin & Lindström, 2010).

Note: All selected articles exceptK. Mueller et al. (2015),Park et al. (2013), andSalimpoor et al. (2013)included self-ratings to check that emotions had indeed been induced through music.

### 3.2 Brain activations evoked by listening to music (40 studies)

The ALE analysis identified one large cluster for the general effect of music-evoked emotions. The list of peak coordinates and MNI coordinates can be found inTable 2(see also the peaks of activations of the clusters inSupplementary Table 2and the contrasts contributing to the clusters inSupplementary Table 3).

**Table 2.** Peak coordinates and anatomical structures activated by listening to emotional music.

Cluster	Brain region	ALE	Z-value	x	y	z	Brodmann area
*1*	R Amygdala	0.055	7.7	20	−8	−16	−
	R Superior Temporal Gyrus	0.046	6.8	50	−20	6	13
	L Superior Temporal Gyrus	0.040	6.1	−52	−4	−6	22
	R Superior Temporal Gyrus	0.040	6.1	52	−12	4	22
	L Lentiform Nucleus Putamen	0.038	5.9	−14	10	−2	−
	L Amygdala	0.037	5.8	−22	−14	−16	−
	L Superior Temporal Gyrus	0.036	5.7	−54	−18	4	41
	R Medial Frontal Gyrus	0.033	5.3	6	54	−8	10
	L Superior Temporal Gyrus	0.032	5.2	−38	−30	10	41
	L Anterior Cingulate	0.031	5.1	−2	36	−8	24
	R Superior Temporal Gyrus	0.031	5.0	56	−2	−6	22
	R Substantia Nigra	0.030	5.0	12	−28	−8	−
	R Putamen	0.028	4.7	28	10	10	−
	R Caudate	0.028	4.7	12	8	−2	−
	L Superior Temporal Gyrus	0.027	4.6	−56	−36	12	42
	L Thalamus	0.026	4.4	2	−18	4	−
	L Insula	0.025	4.3	−34	2	12	13
	R Culmen	0.024	4.2	6	−34	−12	−
	L Claustrum	0.024	4.2	−28	14	12	−
	L Substantia Nigra	0.023	4.1	−8	−28	−12	−
	R Caudate	0.023	4.1	14	30	−10	−
	L Insula	0.021	3.8	−34	22	0	13
	R Claustrum	0.021	3.8	34	4	16	−
	L Insula	0.021	3.8	−36	26	12	13
	L Insula	0.021	3.8	−46	12	2	13
	R Insula	0.020	3.6	46	14	−8	13
	R Insula	0.020	3.6	42	−8	10	13
	R Claustrum	0.019	3.5	36	−8	10	−
	L Anterior Cingulate	0.019	3.5	0	16	−8	25
	R Putamen	0.018	3.3	18	8	−14	−
	L Thalamus	0.017	3.2	−20	−10	10	−
	L Anterior Cingulate	0.016	3.1	−8	48	−2	32
	R Thalamus	0.016	3.0	8	−8	−6	−
	R Inferior Frontal Gyrus	0.016	3.0	34	34	−6	47
	L Sub-Gyral	0.015	2.9	−42	−2	−16	−
	L Caudate	0.015	2.8	−16	8	16	−
	R Caudate	0.014	2.8	10	8	12	−
	R Inferior Frontal Gyrus	0.014	2.8	40	8	−18	13
	L Medial Frontal Gyrus	0.014	2.7	0	54	4	10
	L Putamen	0.013	2.6	−22	−2	8	−
	L Thalamus	0.013	2.6	0	−4	6	−
	R Claustrum	0.011	2.3	32	22	2	−
	R Insula	0.011	2.2	48	6	16	13
	R Inferior Frontal Gyrus	0.011	2.2	30	26	−14	47
	R Superior Temporal Gyrus	0.010	2.0	66	−20	−4	−
	L Hippocampus	0.010	1.9	−34	−32	−10	−
	L Inferior Frontal Gyrus	0.010	1.8	−44	24	−6	47

**Fig. 3.**
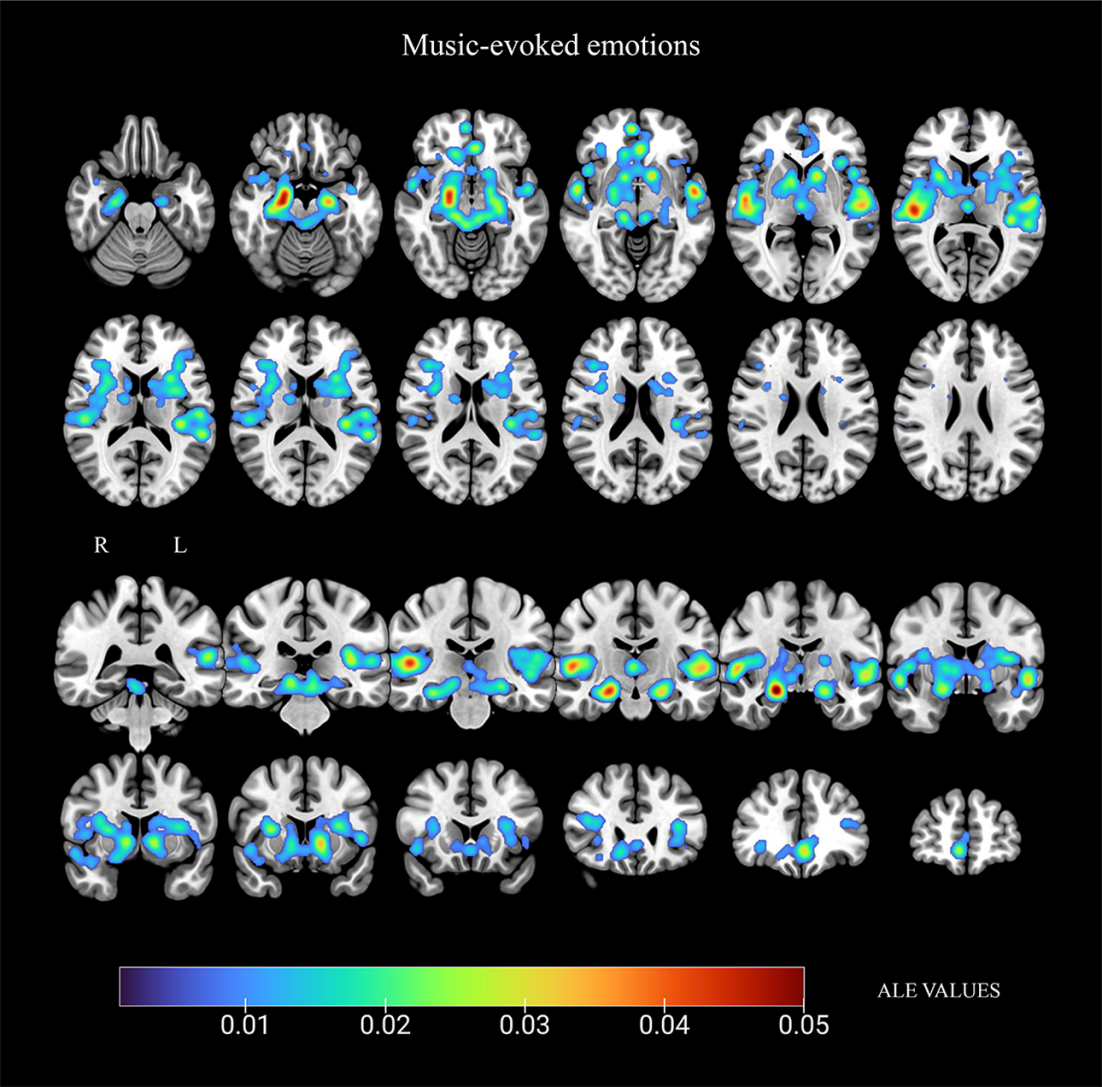
Results from ALE meta-analysis of brain regions active during music-evoked emotions. Radiological convention in coronal slices: R (right) and L (left). Gradient of the activation peaks represented according to their ALE value.

The large cluster included peaks in multiple cortical and subcortical regions. Specifically, bilateral areas in the superior temporal gyrus, inferior frontal gyrus, the middle temporal gyrus, amygdala, prefrontal cortex, substantia nigra, body of caudate, insula, and thalamus were active. Additionally, peaks were identified within the left primary auditory cortex, anterior cingulate, dorsolateral cingulate area, hippocampus, putamen, and caudate head (seeTable 2;Fig. 3).

### 3.3 Brain activity evoked by unpleasant contrasted to pleasant music (15 studies)

For brain activations specific to unpleasant relative to pleasant music listening, our analysis found one large cluster (seeTable 3; see also the contrasts contributing to these clusters inSupplementary Table 5). Specifically, activations were found in the right parahippocampal gyrus, amygdala, culmen, insula, and the inferior frontal gyrus (seeFig. 4). Contrast analyses and conjunction analyses of these contrasts can be found in Supplementary Material (seeSupplementary Fig. 1;Supplementary Tables 7, 8, and 9).

**Table 3.** Peak coordinates and anatomical structures while listening to unpleasant > pleasant music

Cluster	Brain regions	ALE	Z-value	x	y	z	Brodmann area
*1*	R Parahippocampal Gyrus	0.015	4.7	22	−26	−16	35
	R Amygdala	0.0145	4.5	22	−6	−16	−
	R Culmen	0.010	3.4	10	−32	−8	−
	R Parahippocampal Gyrus	0.009	3.4	18	−36	−4	30
	R Insula	0.007	2.9	44	16	−8	13
	R Inferior Frontal Gyrus	0.007	2.7	34	8	−16	13

**Fig. 4.**
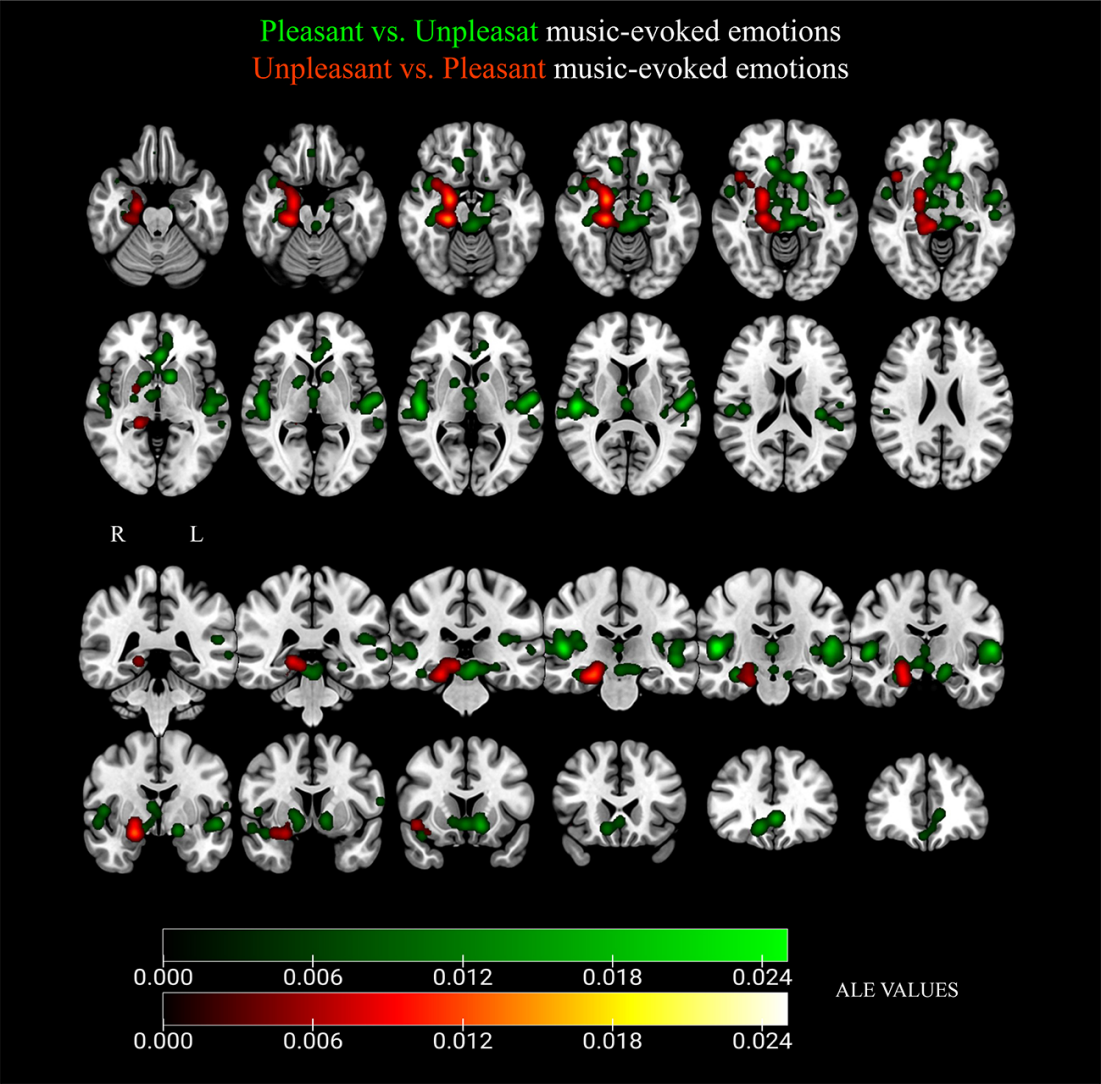
Results from ALE meta-analysis of brain regions active during listening to pleasant (compared with unpleasant) music (green) and unpleasant (compared with pleasant) music (red). Radiological convention in coronal slices: R (right) and L (left). Gradient of the activation peaks represented according to their ALE value.

### 3.4 Brain activity evoked by pleasant contrasted to unpleasant music (17 studies)

The ALE analysis of brain activation during pleasant relative to unpleasant music identified three clusters (seeTable 4; see also the contrasts contributing to these clusters inSupplementary Table 6). Specifically, clusters were found bilaterally in the superior temporal gyrus, the insula, and the thalamus. Peaks of activation were also identified in the right hippocampus, caudate head, orbitofrontal cortex, and the primary auditory cortex, as well as in the left amygdala and anterior cingulate cortex (seeFig. 4). Contrast analyses and conjunction analysis of these contrasts can be found in Supplementary Material (seeSupplementary Fig. 1;Supplementary Tables 7, 8, and 9). Additional analysis exploring differences in brain activation of the different model of emotion (dimensional & discrete) for pleasant music can be found in Supplementary Material (seeSupplementary Figs. 2 and 3;Supplementary Tables 10, 11, and 12).

**Table 4.** Peak coordinates and anatomical structures while listening to pleasant > unpleasant music.

Cluster	Brain region	ALE	Z-value	x	y	z	Brodmann areas
*1*	L Lentiform Nucleus	0.021	5.0	−12	10	−6	−
	L Anterior Cingulate	0.018	4.5	−2	30	−2	−
	R Caudate	0.017	4.4	12	28	−10	−
	R Parahippocampal Gyrus	0.015	4.1	24	−14	−18	28
	L Culmen	0.015	4.0	−6	−30	−10	−
	R Caudate Head	0.015	4.0	12	8	−4	−
	L Amygdala	0.014	3.8	−18	−6	−14	−
	R Hippocampus	0.01	3.7	34	−20	−16	−
	L Substantia Nigra	0.012	3.5	−14	−26	−12	−
	L Anterior Cingulate	0.012	3.5	0	16	−8	25
	L Thalamus	0.012	3.5	2	−18	8	−
	R Thalamus.	0.012	3.5	6	−10	−6	−
	R Medial Globus Pallidus	0.010	3.0	14	0	−12	−
	L Thalamus	0.010	3.0	0	−8	2	−
	R Hippocampus	0.010	3.0	40	−28	−16	−
	R Thalamus	0.010	3.0	6	−4	8	−
	L Mammillary Body	0.010	2.9	−2	−10	−10	−
	L Anterior Cingulate	0.009	2.9	−12	40	2	32
	R Substantia Nigra	0.009	2.8	14	−28	−10	−
	L Anterior Cingulate	0.008	2.7	−10	48	−2	32
	R Medial Frontal Gyrus	0.008	2.6	2	38	−18	11
	L Parahippocampal Gyrus	0.007	2.5	−26	−32	−10	27
	L Anterior Cingulate	0.007	2.3	−4	38	−12	32
*2*	L Superior Temporal Gyrus	0.022	5.2	−58	−12	6	22
	L Superior Temporal Gyrus	0.018	4.6	−50	−18	6	13
	L Superior Temporal Gyrus	0.015	4.1	−50	−22	−2	−
	L Superior Temporal Gyrus	0.014	3.9	−52	−4	−8	22
	L Insula	0.014	3.8	−34	−26	16	13
	L Superior Temporal Gyrus	0.009	2.9	−46	−34	16	41
	L Claustrum	0.009	2.8	−38	−18	6	−
	L Middle Temporal Gyrus	0.008	2.8	−62	−36	2	22
	L Precentral Gyrus	0.008	2.7	−60	2	12	6
	L Superior Temporal Gyrus	0.008	2.7	−54	−38	14	41
*3*	R Superior Temporal Gyrus	0.030	6.3	50	−20	8	13
	R Insula	0.011	3.2	48	0	4	13
	R Insula	0.010	3.1	38	−22	18	13
	R Inferior Frontal Gyrus	0.010	3.1	40	8	−16	13
	R Superior Temporal Gyrus	0.010	3.1	54	−2	−6	22
	R Superior Temporal Gyrus	0.010	3.0	62	−26	6	22
	R Superior Temporal Gyrus	0.009	3.0	66	−26	10	42
	R Claustrum	0.009	3.0	34	−24	10	−

### 3.5 Brain activity evoked by emotional music (pleasant and unpleasant) contrasted to neutral music (eight studies)

**Fig. 5.**
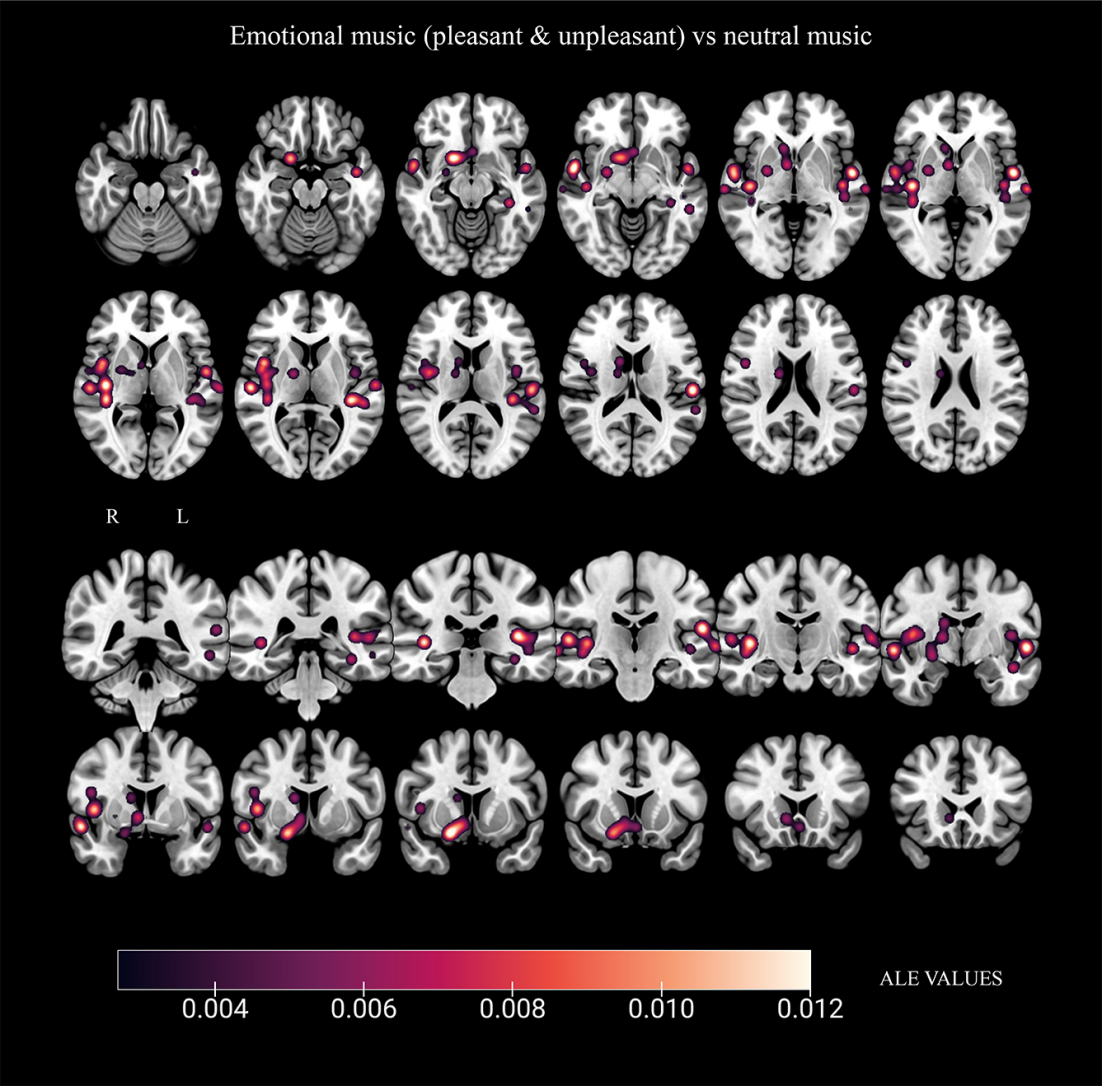
Results from ALE meta-analysis of brain regions active during listening to emotional music (pleasant & unpleasant) contrasted to neutral. Radiological convention in coronal slices: R (right) and L (left). Gradient of the activation peaks represented according to their ALE value.

The analysis for brain activations during the listening of emotional music compared with neutral identified two clusters. These clusters included peaks of activation in the right putamen, claustrum, caudate, globus pallidus, thalamus, and secondary motor cortex, as well as in the left hippocampus and anterior cingulate. Additionally, clusters were found bilaterally in the superior temporal gyrus, primary auditory cortex, and insula (seeTable 5;Fig. 5).

**Table 5.** Peak coordinates and anatomical structures while listening to pleasant and unpleasant > neutral music.

Cluster	Brain regions	ALE	Z-value	x	y	z	Brodmann area
*1*	R Putamen	0.015	4.5	18	10	−14	−
	R Claustrum	0.014	4.4	40	−16	2	−
	R Superior Temporal Gyrus	0.014	4.4	56	0	−8	22
	R Superior Temporal Gyrus	0.013	4.1	56	−20	6	41
	R Caudate	0.013	4.0	40	−30	4	−
	R Insula	0.012	3.9	44	4	6	13
	R Superior Temporal Gyrus	0.012	3.9	56	−6	−4	22
	R Insula	0.010	3.4	40	−6	10	13
	R Superior Temporal Gyrus	0.010	3.4	66	−20	−4	−
	R Lateral Globus Pallidus	0.008	3.0	24	−4	−6	−
	R Caudate	0.008	3.0	6	2	−2	−
	R Putamen	0.007	3.0	26	−2	−2	−
	R Thalamus	0.007	2.9	16	−6	8	−
	L Anterior Cingulate	0.007	2.8	−2	16	−10	25
	R Precentral Gyrus	0.007	2.8	46	4	20	6
	R Caudate	0.007	2.7	10	18	−2	−
	R Caudate	0.007	2.7	14	−6	20	−
	R Caudate	0.007	2.6	12	6	16	−
*2*	L Superior Temporal Gyrus	0.014	4.4	−54	−4	0	22
	L Postcentral Gyrus	0.014	4.2	−56	−22	16	40
	L Transverse Temporal Gyrus	0.013	4.2	−38	−30	8	41
	L Superior Temporal Gyrus	0.010	3.6	−52	0	−10	22
	L Insula	0.010	3.6	−46	−16	−2	13
	L Superior Temporal Gyrus	0.010	3.5	−68	−20	0	22
	L Sub-Gyral	0.010	3.4	−44	−4	−18	21
	L Transverse Temporal Gyrus	0.009	3.3	−62	−16	8	42
	L Superior Temporal Gyrus	0.008	3.2	−50	−34	8	22
	L Hippocampus	0.008	3.2	−36	−32	−10	−
	L Superior Temporal Gyrus	0.007	2.8	−60	−40	14	22
	L Sub-Gyral	0.007	2.8	−48	−28	−4	21
	L Insula	0.007	2.7	−42	−2	10	13
	L Insula	0.007	2.7	−42	−6	10	13
	L Middle Temporal Gyrus	0.007	2.7	−54	−38	−8	20

## 4 Discussion

The present ALE meta-analysis investigated brain activations during listening to emotional music and sought to reveal specific activation as a function of hedonic valence and arousal of the music stimuli. Overall, this meta-analysis revealed peaks of activation and clusters across numerous cortical and subcortical regions related to emotional processing. Additionally, findings revealed that some areas are differentially activated depending on the affective dimensions of hedonic valence and arousal.

### 4.1 Music-evoked emotions

For the overall effect of music-induced emotion (general analysis without considering the affective dimensions of hedonic valence or arousal), findings revealed clusters of activation in cortical and subcortical regions such as the auditory cortex, amygdala, striatum, insula, thalamus, hippocampus, and anterior cingulate cortex. This network largely aligns with previous meta-analyses (Koelsch, 2014,^2020^). In addition, the current analyses suggest that emotional music perception activates the insula, a region crucial for coordinating sensory, emotional, motivational, and cognitive functions (Molnar-Szakacks & Uddin, 2022). Previous research indicates that the insula is connected to limbic regions, as well as to posterior parietal, inferior frontal, and superior temporal cortex (Augustine, 1996). Interestingly, the inferior frontal gyrus is the frontal component of the human mirror neuron system (Molnar-Szakacs & Overy, 2006). The mirror neuron system allows individuals to understand the meaning and intention of a communicative signal by evoking a representation of that signal in the perceiver’s brain (Molnar-Szakacs & Overy, 2006). This mechanism has been shown to respond to auditory stimuli, such as music. Therefore, our findings may support the hypothesis that the insula functions as a neural relay station, connecting the human mirror neuron system (via the inferior frontal gyrus) with the limbic system during music listening. This may help explain the unique capacity of music to communicate meaning and evoke human affect.

Taken together, our findings align with well-established associations of insula involvement in affective processing (Barrett & Wager, 2006;Murphy et al., 2003;Phan et al., 2002), as well as in music perception more broadly (Blood & Zatorre, 2001;Trost et al., 2012). The engagement of the insula during music-evoked emotions contrasts with the results obtained byKoelsch (2020), who found activation of the secondary somatosensory cortex rather than the insula. This divergence might be explained by the fact that the secondary somatosensory cortex extends medially into the superior–posterior insula, which could lead to confusion between these two regions (Koelsch, 2020). Thus, what appears as activation in the secondary somatosensory cortex in some studies may, in fact, be insular activation, underscoring the complex relationship between these adjacent brain areas.

### 4.2 Brain activations as a function of hedonic valence and arousal

The current content-specific analyses also revealed different clusters as a function of the affective valence and arousal of music stimuli. Firstly, we observed that the amygdala was activated during the listening of both unpleasant and pleasant music, but, interestingly, we observed indications of a hemispheric bias depending on the valence of the stimulus, with the right amygdala showing relatively greater activity during unpleasant music perception, and the left amygdala showing relatively greater activity during pleasant music perception. There are not yet enough studies to provide the statistical power needed to directly contrast pleasant and unpleasant (vs. neutral) music-induced emotions, and much prior research has established bilateral amygdala activation during the processing of fearful (LeDoux, 2000;Murphy et al., 2003;Phan et al., 2002) and reward stimuli (Barrett, 2006;Janak & Tye, 2015). It is relevant to note that here we included direct contrasts not only between fear and other specific emotions (e.g., fear vs. joy) but also including other unpleasant forms such as dissonance or dislike, which could suggest that the significant activation of the amygdala is associated with negative mood in general. Following the hypothesis of the dimensional model of emotions, as well as findings obtained in this meta-analysis, amygdala activation relates strongly to the salience of the stimuli (Lang & Bradley, 2013;Sabatinelli et al., 2005). Nevertheless, when considering the rated arousal of the music stimuli, we did not identify activation of the amygdala, suggesting the importance of the hedonic valence of some kind to engage this structure. Interestingly, a recent meta-analysis that sought to investigate brain activation during food-induced pleasure and rewarding music suggested that the amygdala was specifically activated during food-induced pleasure but not during music-induced pleasure (Mas-Herrero et al., 2021), which contrasts with our current results. This finding byMas-Herrero et al. (2021)is surprising given the extensive literature demonstrating the engagement of the amygdala in processing emotional stimuli, particularly in music-induced emotions. One possible explanation for the divergence betweenMas-Herrero et al. (2021)meta-analysis and our findings may lie in the contrasts included in both studies. Specifically, while our study only included contrasts between pleasant and unpleasant music,Mas-Herrero et al. (2021)included a broader set of contrasts (e.g., favourite vs. standard; popular music vs. notes clips or positive correlations with the amount of money willing to pay). Overall, our data clearly show that emotional music induces activation in the amygdala, supporting the potential use of music-based clinical interventions, especially in the treatment of affective disorders, which are often linked to amygdala dysfunctions emotional processing (Koelsch et al., 2010).

Our valence-specific analysis showed that activation of bilateral anterior cingulate cortex (ACC) appeared to be specifically associated with pleasant emotions induced by music. This result is generally consistent with prior research that demonstrates greater activation of this region during chill-inducing (highly pleasant) music (Blood & Zatorre, 2001). However, this finding does not align with other work that implicates the ACC in the processing of negative emotion states (Etkin et al., 2011;Shackman et al., 2011;Tikàsz et al., 2016). Moreover, a recent neuroimaging meta-analysis of music familiarity (Freitas et al., 2018) identified the right anterior cingulate cortex as active during unfamiliar, compared with familiar music listening. Since unfamiliar music is usually rated as less preferred, and less preferred stimuli are typically rated as less pleasant (Fuentes-Sánchez et al., 2022), this finding is consistent with the idea of the preferential processing of negative/unpleasant emotions in the ACC. Surprisingly, in the current analysis of music arousal, we identified greater activation of this region during emotional versus neutral music listening, suggesting that the ACC is driven by emotional intensity, not valence. These results are consistent with recent work that has identified activation in ACC specifically during listening to pleasant music rated as activating, but not during less activating unpleasant music (Fuentes-Sánchez et al., 2021a,2021b).

Our valence-specific analysis also revealed clusters of activations in the dorsal striatum (i.e., caudate, lentiform nucleus) during the processing of pleasant emotions. These results replicate prior findings (Salimpoor et al., 2011,^2013^;Trost et al., 2012) in that experiencing pleasure during music listening activates the reward network. In fact, prior work showed that the activation of the dorsal and ventral striatum was proportional to the reward value of the stimuli (Salimpoor et al., 2013). A more recent study showed that the dopaminergic system was recruited during rewarding music perception (Ferreri et al., 2019). Since the dorsal striatum is part of the reward system, the activation of the caudate region during music-evoked emotions might suggest that musical stimuli might have similar properties to other rewarding experiences, such as food, sex, or winning money, which have been shown to activate these regions (Belfi & Loui, 2019;Mas-Herrero et al., 2021;Small et al., 2001,^2003^). These findings further support the use of music for psychological treatment in pathologies showing underactivation of the striatum, such as anhedonia (e.g.,Borsini et al., 2020) or major depressive disorder (e.g.,Forbes et al., 2009). However, despite mounting evidence for an involvement of the ventral striatum—particularly the nucleus accumbens—in pleasant music perception (Koelsch, 2014;Mantione et al., 2014;Salimpoor et al., 2013;Zatorre & Salimpoor, 2013), we did not find reliable clusters of activation in this subcortical structure. This apparent lack of replication might be explained by the small size of the reward-specific nucleus accumbens (NAcc), and the tendency for co-activity of surrounding subcortical structures during music perception and appreciation, which can lead to variable reporting in coordinate location across individual studies. Depending on the design of the particular experiment, this location discrepancy can become worse when long-duration music pieces lead to increasingly large BOLD signal clusters as a result of elevated venous blood oxygenation. For example, if a study identified a large cluster of activity that included much of basal ganglia, the reported cluster coordinate might be centered many millimeters away, perhaps in a more medial location than NAcc, although the cluster in fact contains NAcc. In fMRI meta-analyses, this study might contribute one midline coordinate from the center of mass of that large cluster, depending upon the reporting choices made by the authors. Indeed, this effect is evident in theMas-Herrero et al. (2021)meta-analysis, which identified a large, single cluster at the midline of the brain during music reward, with bilateral NAcc included at the lateral edges of this cluster. During food reward, large bilateral clusters were centered on putamen, with NAcc again appearing on the boundaries of this cluster, in this case the medial edges. In neither case was there clear, bilateral activity centered only in NAcc, as can be seen in meta-analyses of monetary reward (Sescousse et al., 2013). In our current meta-analyses, the large clusters were broken into as many subregions as were available, with distinct coordinates, and named according to the ALE output. Another possible contributor to the lack of clear NAcc activity here is the wide range of pleasant and unpleasant music chosen as stimuli across the studies. For example, the Salimpoor and Martinez-Molina studies (Salimpoor et al., 2011,^2013^) specifically targeted extremely pleasant music that reliably induced chills in their subjects. This chill-inducing music led to the greatest activity in NAcc, while other pleasant music showed weaker effects. Similarly, a recent fMRI meta-analysis of humor perception did not identify clear evidence of NAcc activity (Farkas et al., 2021), despite the common belief that this structure contributes strongly to humor processing.

The present meta-analyses also identified activation of bilateral thalamus while listening to pleasant music, as well as while listening to music in general (without considering the hedonic valence). Interestingly, greater activation of the right thalamus was found during arousing (pleasant and unpleasant) relative to neutral music perception, consistent with emotional scene perception studies (Anders et al., 2004;Frank & Sabatinelli, 2014). To this respect, a number of previous studies (Blood & Zatorre, 2001;Klepzig et al., 2020;Salimpoor et al., 2011) showed that this region was involved during chill response processing (piloerection associated with a positive emotional response). These findings also align with those obtained using peripheral physiological measures (Fuentes-Sánchez et al., 2021b), as an enhanced reactivity of the sympathetic nervous system, a system related to emotional arousal, was found during listening to pleasant but also unpleasant music. Interestingly, in such work, it was found that the sympathetic response was enhanced during the listening of pleasant music, which, in turn, was rated as more arousing, in comparison with the processing of unpleasant music (Fuentes-Sánchez et al., 2021b).

The hippocampus was also found to be active during emotions induced by music, and especially while listening to pleasant music. The role of the hippocampus in emotional processing has been met with mixed findings in the literature, with studies showing activations while listening to unpleasant music (Koelsch et al., 2006;Mitterschiffthaler et al., 2007) and pleasant music (Koelsch et al., 2010;Trost et al., 2012). The hippocampus does contribute to the processing of emotions (Koelsch, 2014,^2020^), which is in line with the findings from the current study. In fact, our results support the hypotheses proposed byKoelsch (2014,^2020^), highlighting the hippocampus as an important region for the generation of pleasant emotions (Koelsch, 2020;Koelsch et al., 2010). Specifically,Koelsch (2020)claims that hippocampus activation is strongly associated with attachment-related emotions and social bonding elicited by music. In our analysis, pleasant music encompasses different emotions such as joy, pleasantness, wonder, tenderness, or liking, emotions that have been demonstrated to activate regions such as the prefrontal cortex or the insula, which are brain areas related with social bonding (Greenberg et al., 2021). Therefore, these results suggest that music listening, and particularly listening to pleasant music, may provide an effective means to facilitate social connection; as such, it could represent an effective intervention to social isolation, which affects a large and rapidly increasing percentage of the population, especially in the elderly (Fakoya et al., 2020).

Furthermore, our findings showed that the parahippocampal gyrus (PHG) revealed activation during both pleasant and unpleasant emotional music. The activation of the PHG has been typically associated with the recognition of emotions and retrieval of strong emotional memories (Blood et al., 1999;Koelsch et al., 2006). Therefore, findings obtained in this work may demonstrate that the PHG is involved in the recognition of music emotions, independent of their affective valence, in contrast to previous neuroimaging studies that associated the involvement of this regions in networks responsive to specifically unpleasant emotions (Blood et al., 1999;Koelsch et al., 2006). When considering the arousal effect, no activations were found in this region, which suggest that the activation of the parahippocampal gyrus is more related to hedonic valence. In this line, meta-analyses of neuroimaging studies on emotion processing in general (Wager et al., 2003), and emotional face processing (Fusar-Poli et al., 2009) in healthy subjects, revealed bilateral activations of the parahippocampal gyri across valence conditions.

The activation of the hippocampal and parahippocampal regions during music listening may also be related to the evocation of autobiographical memories (Belfi et al., 2016). Prior research suggests that the engagement of the hippocampus, alongside the limbic system, plays a significant role in music-evoked responses that are influenced by memory (Ferreri & Rodríguez-Fornells, 2022). Specifically, previous studies have shown that musical pleasure can enhance memory, a process modulated by dopaminergic activity and individual differences in music reward sensitivity (Ferreri & Rodríguez-Fornells, 2022). The finding that listening to music improves memory has clinical implications, especially in stimulating episodic and autobiographical memories in clinical populations such as those with Alzheimer’s disease.

Furthermore, our valence-specific meta-analysis revealed activation in auditory cortical regions such as the superior temporal gyrus (STG) and the inferior frontal gyrus (IFG) during the processing of emotions induced by music, particularly in the context of pleasant emotions. This engagement of auditory areas in music-induced emotions aligns with previous meta-analysis byKoelsch (2020), suggesting that the auditory cortex is not only crucial for musical cognition (Albouy et al., 2018;Coffey et al., 2016), but also for emotion processing (Koelsch, 2014,^2020^). Similarly, our results support the findings byMas-Herrero et al. (2021), which highlighted the involvement of these structures in music-induced pleasure. Notably, prior fMRI studies demonstrated that increased functional connectivity between the STG and the ventral striatum predicts the reward value of music (Salimpoor et al., 2013).

### 4.3 Limitations and future directions

As is any meta-analysis, it is important to highlight the great variability between studies at both conceptual and methodological levels. At the conceptual level, some studies focused on the discrete emotion perspective, such as happiness, fear, or sadness (e.g.,Aubé et al., 2015;Bogert et al., 2016;Brattico et al., 2011), whereas other studies focused on the dimensional approach, considering broader concepts such as pleasantness/unpleasantness or arousal (e.g.,Bravo et al., 2020;Chapin et al., 2010;Flores-Gutiérrez et al., 2007). At the methodological level, studies selected for this meta-analysis presented different types of music stimuli (e.g., instrumental, film scores, popular music, dissonant/consonant music), as well as different durations of music excerpts, ranging from 2 seconds to more than 1 minute. These methodological divergences could complicate the consistency and interpretation of the meta-analyses. Specifically, the studies included in this meta-analysis focused only on healthy controls, excluding clinical populations. However, among the healthy control groups, some studies included both musicians and non-musicians (Matthews et al., 2020;Park et al., 2014;Zhou et al., 2022), which could also influence the results due to the potential impact of musicianship on the neural correlates of music processing (Hyde et al., 2009;Palomar-García et al., 2017). Furthermore, the study performed byMartínez-Molina et al. (2016), which included participants with music anhedonia (n = 15), was also part of this meta-analyses. Since music anhedonia is related to the reduced activity in the NAcc, the inclusion of these participants implies a limitation of this meta-analysis, as it could affect the observed results related to reward processing.

A second limitation of the current work is related to the sub-analyses, in which broad categories of emotional content were combined. For example, in the analysis of brain activity evoked by pleasant music, we included contrasts such as “joy > fear,” “like > dislike,” “major > minor,” “pleasant > unpleasant.” Future meta-analyses in the field might differentiate these categorical approaches (e.g., investigating whether there are differences between “joy > fear” and “pleasant > unpleasant” in the neural correlates). Likewise, within the sub-analyses of pleasant and unpleasant processing, we considered emotions varying in terms of hedonic valence but also in arousal. As more studies of pleasant and unpleasant music perception are published, future work may have enough statistical power to more directly evaluate the (pleasant vs. neutral) versus (unpleasant vs. neutral) contrast. As the field of music therapy expands, future meta-analyses might also investigate altered brain activations during music listening in patients suffering from disordered emotional reactivity, such as depression or anxiety.

## 5 Conclusions

The present study showed significant clusters of activations in a wide range of cortical and subcortical regions during music-evoked emotions, expanding and refining past meta-analyses (Koelsch, 2014,^2020^). This work was the first to systematically compare the neural correlates of pleasant and unpleasant emotions induced by music stimuli, and to consider the effect of music intensity, separate from valence. The results obtained in this study showed that subsets of overlapping structures were specifically activated as a function of valence and arousal of the stimuli. Particularly, whereas the ACC, dorsal striatum, and thalamus were varied with arousal, amygdala activation is more sensitive to hedonic valence. Taken together, these findings provide useful information about the brain areas involved in emotional music perception. From a clinical viewpoint, these results could open an avenue toward the development of standardized music therapy protocols as a tool to evoke and regulate emotions, especially in affective or neurodegenerative disorders characterized by anomalies in emotional processing, reactivity, and regulation, such as depression, anxiety, or dementia.

## Supplementary Material

Supplementary Material

Meta-analysis_Data availability

## Data Availability

Data are available as Supplementary Material.

## Footnotes

† We included some studies that use clinical populations, but in the ALE analyses we only considered the control group (healthy population).

‡ The activation likelihood estimation (ALE) consists in estimating the likelihood of overlap between the activation of the coordinates of the foci selected of the included studies in the meta-analysis based on their modeling as probability distributions centered on the respective coordinates. These results are then compared with a null distribution computed over several permutations, in our case 1000. Essentially, the clusters reported in several studies (through the foci) are compared with a distribution of clusters generated at random, and then the clusters are considered significant if they are greater than the greatest 5% of the randomly generated clusters.
