# Supplementary material for: On joy and sorrow: Neuroimaging meta-analyses of music-induced emotion
Source: Imaging Neurosci (Camb). 2025 Jan 16;3:imag_a_00425. doi: 10.1162/imag_a_00425 (PMC12319861; doi:10.1162/imag_a_00425)
Supplement: Supplementary Material [file imag_a_00425-supp.pdf]

# On joy and sorrow: Neuroimaging meta analyses of music induced emotion

**Authors:** Nieves Fuentes-Sánchez[1,#], *Alejandro Espino-Payá[2,3,#], Sabine Prantner[4,5], Dean Sabatinelli[6], M. Carmen Pastor[4,#] & Markus Junghöfer[2,3,#,*]*

[1]Departamento de Psicología, Universidad de Castilla-La Mancha, Facultad de Medicina, Albacete, Spain

[2]Institute for Biomagnetism and Biosignalanalysis, University of Münster, Münster, Germany

[3]Otto Creutzfeldt Center for Cognitive and Behavioral Neuroscience, University of Münster, Münster, Germany.

[4]Departamento de Psicología Básica, Clínica y Psicobiología, Universitat Jaume I, Facultad de Ciencias de la Salud, Castelló de la Plana, Spain

[5]Institute of Psychology, Clinical Psychology and Psychotherapy in Childhood and Adolescence, University of Osnabrueck, Osnabrueck, Germany

[6]Department of Psychology, University of Georgia, Athens, United States

*Corresponding author. Institute for Biomagnetism and Biosignalanalysis. Malmedyweg 15. D-48149 Münster. email: markus.junghoefer@uni-muenster.de

# equal contribution

## SUPPLEMENTARY MATERIAL

**Supplementary Table 1.** Studies included in the general analysis of the meta-analysis

| Study | Modality | Nr. of Subjects | Age (M, SD) | Nr. of Stimuli | Stimuli Duration | Type of Stimuli | Emotion Elicited | Theoretical Approach |
|---|---|---|---|---|---|---|---|---|
| **Aubé et al., 2015** | fMRI | 47 | M = 26.4; SD = 4.8 | 60 | Average 1.47s | Novel instrumental clips selected from a previous validated dataset | Fear, sadness, happiness, and peacefulness (neutral condition) | Discrete |
| **Altenmüller et al., 2014** | fMRI | 18 | M = 28.7; SD = 8.7 | 60 | 10s | Film music | Less positive and very positive | Dimensional |
| **Berthold-Losleben et al., 2018** | fMRI | 32 (16 females) | M = 25, SD = 3.30 | 12 | 16s | Classical music | Pleasant and neutral | Dimensional |
| **Brattico et al., 2016** | fMRI | 29 (15 females) | M = 23.9, SD = 3.1 | 64 | 18s | Different genres | Happy and sad | Discrete |
| **Blood et al., 1999** | PET | 10 (5 females) | - | 6 | 13s each fragment | Consonant or dissonance music | Consonance and dissonance | Dimensional |
| **Blood & Zatorre, 2001** | fMRI | 10 (5 females) | Age range 20-30 | 4 | 90s | Selected classical music; two baseline conditions; and silence) | Selected music vs. control music (selected by other participants) | - |
| **Chapin et al., 2010** | fMRI | 14 (9 females) | - | 2 musical excerpts and 1 baseline silence | 3 min and 36s | Classical music | Arousal and valence | Dimensional |
| **Daly et al., 2019** | fMRI | 21 | M = 24, SD = 2.6 | 40 (36 generated music; 4 classic) | 40s for generated music; classical music (127s, 15s, 170s, 158s) | Classical music and generated music | Arousal and valence | Dimensional |
| **Flores-Gutiérrez et al., 2007** | fMRI | 6 | M = 25; SD = 3.05 | 3 | 30s | Classical music | Pleasant and unpleasant | Dimensional |
| **Jeong et al., 2011** | fMRI | 15 | M = 22.8, SD = 3.4 | 2 | 30s | Classical music | Happy and sad | Discrete |
| **Koelsch et al., 2021** | fMRI | 24 (13 females) | M = 22.79, SD = 3.45 | 12 | 30s | Joy excerpts (soul, jazz, classical music…); fear (from suspense movies and video games) | Joy and fear | Discrete |

| | | | | | | | | |
|---|---|---|---|---|---|---|---|---|
| **Koelsch et al., 2013** | fMRI | 18 | M = 23.78; SD = 3.54 | 24 | 30s | Joy stimuli (classical music, Irish jigs, jazz, reggae, South American and Balkan music), fear music from soundtracks of suspense movies and video games | Joy, fear and neutral | Discrete |
| **Koelsch et al., 2018** | fMRI | 24 | M = 23.3, SD = 3.3 | 24 | 30s | Joy stimuli (classical music, Irish jigs, jazz, reggae, South American and Balkan music), fear music from soundtracks of suspense movies and video games | Joy, fear and neutral | Discrete |
| **Koelsch et al., 2008** | fMRI | 20 (10 females) | Musicians (M = 26.8); Non-musicians (M = 25.6) | 72 | 4s | Chord sequences | Regular chords and irregular chords | - |
| **Koelsch et al., 2006** | fMRI | 11 (5 females) | M = 24.6 | 16 | Average 55s | Joyful pleasant (instrumental dance-tunes); unpleasant (electronically manipulate counterparts of the original tunes) | Unpleasant and pleasant | Dimensional |
| **Khalfa et al., 2005** | fMRI | 13 (5 females) | M = 28 | 34 | 10s | Western classical music | Happy (major mode) and sad (minor mode) | Discrete |
| **Kim et al., 2017** | fMRI | 23 (13 females) | M = 25.9, SD = 2.9 | 100 | 30s | Instrumental tunes including classical music and tango | Dissonant music | Dimensional |
| **Kleipzig et al., 2020** | fMRI | 16 (12 females) | M = 22.3, SD = 3.4 | 12 | 90s | Classical music | Pleasant and unpleasant chills | Dimensional |
| **Kornysheva et al., 2010** | fMRI | 18 | M = 25.1 | 108 | 3s | Auditory musical rhythms (drum sounds generated) | - | - |
| **Lehne et al., 2014** | fMRI | 25 | M = 23.9, SD = 2.9 | 8 | Range between 1min 34s – 2 min 25 s | Piano classical pieces | Tension | Dimensional |
| **Lepping et al., 2016** | fMRI | 20 | M = 28.50; SD = 11.4 | 36 | 10s | Western art music | Positive and negative | Dimensional |
| **Liu et al., 2018** | fMRI | 48 (25 | M = 20.77, | 12 | - | Instrumental music | Valence and arousal | Dimensional |

| | | | | | | | | |
|---|---|---|---|---|---|---|---|---|
| | | females) | SD = 1.87 | | | | | |
| **Martínez-Molina et al., 2016** | fMRI | 45 | Anhedonic participants (M = 21.9, SD = 3.2); Hedonic (M = 20.8, SD = 3.4); Hiper-hedónic (M = 21.3, SD = 5) | 16 | 1min | - | Pleasant and unpleasant | Dimensional |
| **Matthews et al., 2020** | fMRI | 54 | Non-musicians (M = 23.20, SD = 2.45); Musicians (M = 23.76, SD = 2.84) | 36 | 10s | Piano chords | Rhythm, harmony (medium/high) | - |
| **Menon & Levitin, 2005** | fMRI | 13 (7 females) | Age range 19.4-26.6 | - | 23s | Digitalized sound files | Music (pleasant) and scrambled | Dimensional |
| **Mizuno & Sugishita, 2007** | fMRI | 18 | M = 23.17 | 180 | 2s | Tonal music | Major, minor and neutral | - |
| **Mitterschiffhaler et al., 2007** | fMRI | 16 (10 females) | M = 30.18, SD = 5.76 | 20 | 30s | Instrumental from 18th, 19th, and 20th centuty | Happy, sad, and neutral | Discrete |
| **Mueller et al., 2011** | fMRI | 20 (7 females) | M = 26.3, SD = 4 | 50 | 10s | Instrumental tunes | Joyful and dissonant | Dimensional |
| **Mueller et al., 2015** | fMRI | 23 (13 females) | M = 25.9, SD = 2.9 | 80 | 10s | Instrumental tunes | Forward consonant, forward dissonant, backward consonant, backward dissonant | Dimensional |
| **Oetken et al., 2017** | fMRI | 20 (11 women) | M = 26.6, SD = 5.51 | 60 | 35s | Classical music | Happy, sad, and neutral | Discrete |
| **Okuya et al., 2017** | fMRI | 20 (2 females) | M = 31.3, SD = 10.7 | 20 | 30s | | Happy, sad, calm, fear | Discrete |

| | | | | | | | | |
|---|---|---|---|---|---|---|---|---|
| **Park et al., 2013** | fMRI | 12 | M = 20.33, SD = 2.14 | 24 | 21s | Composed for this experiment to express basic emotions | Happiness, sadness, and fear | Discrete |
| **Salimpoor et al., 2013** | fMRI | 19 (10 females) | M = 23.2, SD = 5.1 | 60 | 30s | New music (matched to their preferences) | Music purchased vs. not purchased | Dimensional |
| **Salimpoor et al., 2011** | PET and fMRI | 10 | M = 20.8, SD = 1.9 | - | - | Self-selected music, another's choices | Pleasant and neutral | Dimensional |
| **Shany et al., 2019** | fMRI | 40 (for fMRI 31 in Ligeti; 28 in Glass, and 28 in Mussorgsky) | Ligeti (M = 25.91, SD = 3.71); Glass (M = 25.87, SD = 3.81); Mussorgsky (M = 25.94, SD = 3.75) | 32 | Ligeti 7:51 min; Glass 7:03 min, Mussorgsky 10:57 min | Piano pieces | Highly surprising, low-surprising, and unsurprising | Discrete |
| **Sievers et al., 2021** | fMRI | 20 (11 female) | - | 18 | 3s | Piano melodies | Angry, happy, peaceful, sad, and scared | Discrete |
| **Skouras et al., 2014** | fMRI | 32 | M = 22.93, SD = 2.75 | 24 | 30s | - | Joy, fear, neutral | Discrete |
| **Suzuki et al., 2008** | PET | 13 | - | 96 | 12s | Chord sequences | Consonant and dissonant chords | Dimensional |
| **Tabei, 2015** | fMRI | 17 (10 females) | M = 21.4, SD = 2 | 24 | Average 12.4s | From Vieillard dataset | Happy and sad | Discrete |
| **Trost et al., 2012** | fMRI | 16 (9 females) | M = 29.9, SD = 9.8 | 27 | 45s | Instrumental music | Joy, sadness, tension, wonder, peacefulness, power, tenderness, nostalgia, and transcendence | Discrete |

**Supplementary Table 2**. Peaks of activation in the different clusters for the general meta-analysis

| Cluster | Hemisphere | Lobe | Region | Cell Type |
|---|---|---|---|---|
| *1* | | | | |
| | 49.9% Right Cerebrum | 63.1% Sub-lobar | 22.8% Lentiform Nucleus | 17% Brodmann area 13 |
| | 49% Left Cerebrum | 19.3% Temporal Lobe | 16.8% Insula | 13% Putamen |
| | | 11% Limbic Lobe | 14.2% Superior Temporal Gyrus | 8.8% Brodmann area 22 |
| | | 4.5% Frontal Lobe | 12% Caudate | 6.5% Lateral Globus Pallidus |
| | | | 7.7% Thalamus | 6.5% Brodmann area 41 |
| | | | 6.3% Parahippocampal Gyrus | 6.3% Caudate Head |
| | | | 4.7% Anterior Cingulate | 5.7% Caudate Body |
| | | | 3.6% Transverse Temporal Gyrus | 3% Medial Globus Pallidus |
| | | | 2.8% Claustrum | 2.2% Amygdala |
| | | | 1.8% Precentral Gyrus | 2.1% Brodmann area 32 |
| | | | 1.5% Medial Frontal Gyrus | 1.7% Medial Dorsal Nucleus |
| | | | | 1.6% Hippocampus |
| | | | | 1.6% Brodmann area 10 |
| | | | | 1.5% Brodmann area 24 |
| | | | | 1.4% Brodmann area 28 |
| | | | | 1.2% Brodmann area 34 |
| | | | | 1% Brodmann area 42 |

**Supplementary.** Peaks of activation in the different clusters for the general meta-analysis

1: 133376 mm^3 from (-70,-44,-26) to (70,60,24) centered at (0.4,-4,-.3) with 48 peaks

with a max value of 0.0551 ALE, 8.902671E-15 P, 7.67 Z at (20,-8,-16)

**Supplementary Table 3.** Contrasts contributing to the clusters identified in the general meta-analysis

| Cluster | Nº focis | Contrasts (studies) |
|---|---|---|
| *1* | | |
| | 4 | Koelsch et al., 2013. Joy > Fear |
| | 2 | Koelsch et al., 2018. GLM results of the comparisons between emotion conditions. Joy > fear |
| | 1 | Koelsch et al., 2018. GLM results of the comparisons between emotion conditions. Fear > neutral |
| | 2 | Koelsch et al., 2018. GLM results of the comparisons between emotion conditions. Neutral > fear |
| | 2 | Koelsch et al., 2018. GLM results of the comparisons between emotion conditions. Joy > neutral |
| | 2 | Lehne et al., 2014. Tension (versions with dynamics) > tensión (versions without dynamics) |
| | 4 | Lehne et al., 2014. Tension increase > tension decrease |
| | 1 | Martínez-Molina et al., 2016. Pleasure ratings as a parametric effect |
| | 4 | Skouras et al., 2014. 3 Tesla. Joy > fear |
| | 2 | Skouras et al., 2014. 1.5 Tesla. Joy > fear |
| | 2 | Sievers et al., 2021. Music listening mixed emotion vs. neutral |
| | 5 | Matthews et al., 2020. Medium > High Rhytmic complexity contrast. |
| | 9 | Daly et al., 2019. BOLD co-variation with reported felt affect (but not with movement) during generated music listening task |
| | 6 | Daly et al., 2019. BOLD co-variation with reported felt affect (but not with movement) during classical music listening task |
| | 2 | Jeong et al., 2011. Happy music > sad music |
| | 4 | Kim et al., 2017. Common effect in BOLD for individual diff. in dislike of dissonant music |
| | 1 | Kim et al., 2017. Intersubject correlation in BOLD and rating contrast |
| | 7 | Klepzig et al., 2020. Music with individual pleasant chill > music without chill |
| | 2 | Liu et al., 2018. Fast – Slow |
| | 2 | Liu et al., 2018. Medium – Slow |
| | 3 | Oetken et al., 2017. Musically induced mood (happy vs. sad vs. neutral) X self-evaluation vs. lexical decision making |
| | 2 | Salimpoor et al., 2011. PET Pleasant – neutral |
| | 7 | Salimpoor et al., 2011. fMRI. Pleasant > neutral |
| | 2 | Aubé et al., 2015. Music fear > neutral |
| | 2 | Aubé et al., 2015. Music happiness > neutral |
| | 2 | Aubé et al., 2015. Music (correlations between BOLD signal and intensity) |
| | 11 | Berthold-Losleben et al., 2018. Music positive > neutral |
| | 7 | Brattico et al., 2016. Like > Dislike |
| | 3 | Brattico et al., 2016. Dislike > Like |
| | 3 | Brattico et al., 2016. Happy > Sad |
| | 1 | Brattico et al., 2016. Sad > Happy |
| | 14 | Koelsch et al., 2021. Joy – Fear |
| | 4 | Menon & Levitin, 2005. Scrambled music > Pleasant music |
| | 7 | Mueller et al., 2011. Joyful instrumental tunes > reversed dissonant |
| | 15 | Mueller et al., 2011. Correlation with pleasantness |
| | 3 | Okuya et al., 2017. Happpy |
| | 14 | Okuya et al., 2017. Fear |
| | 6 | Salimpoor et al., 2013. Music purchased vs. music not purchased |
| | 4 | Salimpoor et al., 2013. Music purchased vs. music not purchased (participants who found the music highly rewarding) |
| | 4 | Trost et al., 2012. Correlation with tension |
| | 4 | Trost et al., 2012. Correlation with joy, power, and wonder |
| | 7 | Trost et al., 2012. Correlation with peacefulness, tenderness, nostalgia, and transcendence |
| | 3 | Trost et al., 2012. Correlation with sadness |
| | 6 | Trost et al., 2012. Correlation with ratings of high arousal |
| | 10 | Trost et al., 2012. Correlation with ratings of positive valence |
| | 4 | Lepping et al., 2016. Main effect of valence. Positive > negative |
| | 5 | Mizuno & Sugishita, 2007. Major – neutral |
| | 3 | Mizuno & Sugishita, 2007. Minor – neutral |
| | 1 | Mizuno & Sugishita, 2007. Major – minor |
| | 18 | Park et al., 2013. Happiness vs. control (relaxing music) |
| | 12 | Park et al., 2013. Fear vs. control (relaxing music) |
| | 29 | Shany et al., 2019. Brain activation as a function of surprise level |
| | 2 | Kornysheva et al., 2010. Beautiful vs. not beautiful rhythms |
| | 2 | Chapin et al., 2010. Emotional arousal: experienced vs. inexperienced |
| | 4 | Koelsch et al., 2008. Irregular > regular chords |
| | 4 | Mitterschiffthaler et al., 2007. Happy > neutral music |
| | 4 | Mitterschiffthaler et al., 2007. Sad > neutral music |
| | 2 | Mitterschiffthaler et al., 2007. Sad > neutral music |
| | 2 | Mitterschiffthaler et al., 2007. Neutral > Sad & happy |

| | |
|---|---|
| 1 | Suzuki et al., 2008. Beautiful consonance vs. ugly dissonance |
| 2 | Suzuki et al., 2008. Ugly dissonance vs. beautiful consonance |
| 1 | Suzuki et al., 2008. Minor vs. major |
| 4 | Suzuki et al., 2008. Beautiful minor vs. ugly minor |
| 1 | Suzuki et al., 2008. Beautiful minor vs. beautiful major |
| 1 | Blood et al., 1999. Positive correlation with dissonance |
| 3 | Blood et al., 1999. Negative correlations with ratings of pleasantness |
| 2 | Blood et al., 1999. Positive correlations with ratings of pleasantness |
| 1 | Blood et al., 1999. Negative correlations with ratings of pleasantness |
| 6 | Blood & Zatorre, 2001. Positive correlations with chills intensity |
| 4 | Blood & Zatorre, 2001. Negative correlations with chill intensity |
| 6 | Blood & Zatorre, 2001. Increases in rCBF for subject-selected music minus control music |
| 4 | Blood & Zatorre, 2001. Decreases in rCBF for subject-selected music minus control music |
| 5 | Blood & Zatorre, 2001. Positive correlations with pleasantness |
| 3 | Blood & Zatorre, 2001. Negative correlations with pleasantness |
| 5 | Blood & Zatorre, 2001. Positive correlations with emotional intensity |
| 4 | Blood & Zatorre, 2001. Negative correlations with emotional intensity |
| 5 | Flores-Gutiérrez et al., 2007. Pleasant |
| 13 | Flores-Gutiérrez et al., 2007. Unpleasant |
| 5 | Flores-Gutiérrez et al., 2007. Activation (excitement) |
| 6 | Flores-Gutiérrez et al., 2007. Activation (calmness) |
| 5 | Koelsch et al., 2006. Unpleasant > pleasant |
| 6 | Koelsch et al., 2006. Pleasant > unpleasant |
| 2 | Altenmüller et al., 2014. Positive pieces > less positive pieces |

**Supplementary Table 4.** List of included studies in the sub-analysis of the meta-analysis

| Sub-analysis | Study | Contrast |
|---|---|---|
| *Brain activity evoked by pleasant music* | | |
| | Koelsch et al., 2013 | Joy > fear |
| | Koelsch et al., 2018 | GLM results of the comparisons between emotion conditions. Joy > Fear |
| | Martínez-Molina et al., 2016 | Pleasure ratings as a parametric effect |
| | Skouras et al., 2014 | 3 Tesla. Joy > fear |
| | Skouras et al., 2014 | 1.5 Tesla. Joy > fear |
| | Brattico et al., 2016 | Like > dislike |
| | Koelsch et al., 2021 | Joy – fear |
| | Mueller et al., 2015 | Correlation with pleasantness |
| | Okuya et al., 2017 | Happy |
| | Trost et al., 2012 | Correlation with joy, power, and wonder |
| | Trost et al., 2012 | Correlation with peacefulness, tenderness, nostalgia, and trascendence |
| | Trost et al., 2012 | Correlation with ratings of positive valence |
| | Lepping et al., 2016 | Main effect of valence. Positive > negative |
| | Kornysheva et al., 2010 | Beautiful vs. not beautiful rhythms |
| | Suzuki et al., 2008 | Beautiful consonance vs. ugly dissonance |
| | Blood et al., 1999 | Negative correlations with dissonance |
| | Blood et al., 1999 | Positive correlations with ratings of pleasantness |
| | Blood & Zatorre, 2001 | Positive correlations with chill intensity |
| | Blood & Zatorre, 2001 | Positive correlations with pleasantness |
| | Flores-Gutiérrez et al., 2007 | Pleasant |
| | Koelsch et al., 2006 | Pleasant > unpleasant |
| | Altenmüller et al., 2014 | Positive pieces > less positive pieces |
| *Brain activity evoked by unpleasant music* | | |
| | Koelsch et al., 2013 | Fear > joy |
| | Koelsch et al., 2018 | GLM results of the comparisons between emotion conditions. Fear > joy |
| | Lehne et al., 2014 | Positive correlations with tension |
| | Kim et al., 2017 | Common effect in BOLD for individual diff. in dislike of dissonant music |
| | Brattico et al., 2016 | Dislike > like |
| | Menon & Levitin, 2005 | Scrambled music > pleasant music |
| | Okuya et al., 2017 | Fear |
| | Trost et al., 2012 | Correlation with tension |
| | Trost et al., 2012 | Correlation with ratings of negative valence |
| | Lepping et al., 2016 | Main effect of valence. Negative > positive |
| | Koelsch et al., 2008 | Irregular > regular chords |
| | Suzuki et al., 2008 | Ugly dissonance vs. beautiful consonance |
| | Blood et al., 1999 | Positive correlations with dissonance |
| | Blood et al., 1999 | Negative correlations with ratings of pleasantness |
| | Blood & Zatorre, 2001 | Negative correlations with pleasantness |
| | Flores-Gutiérrez et al., 2007 | Unpleasant |
| | Koelsch et al., 2006 | Unpleasant > pleasant |

| | | |
|---|---|---|
| *Brain activity evoked by emotional music compared to neutral music* | | |
| | Koelsch et al., 2018 | GLM results of the comparisons between emotion conditions. Fear > neutral |
| | Koelsch et al., 2018 | GLM results of the comparisons between emotion conditions. Joy > neutral |
| | Sievers et al., 2021 | Music listening mixed emotion vs. neutral |
| | Salimpoor et al., 2011 | PET Pleasant – neutral |
| | Salimpoor et al., 2011 | fMRI Pleasant > neutral |
| | Aubé et al., 2015 | Music fear > neutral |
| | Aubé et al., 2015 | Music happiness > neutral |
| | Berthold-Losleben et al., 2018 | Music positive > neutral |
| | Mizuno & Sugishita, 2007 | Major – neutral |
| | Park et al., 2013 | Happiness vs. control (relaxing music) |
| | Park et al., 2013 | Fear vs. control (relaxing music) |
| | Mitterschiffthaler et al., 2007 | Happy > neutral music |

**Supplementary.** Peaks of activation in the different clusters for the sub-analysis of unpleasant contrasted to pleasant music

1: 15656 mm^3 from (4,-42,-28) to (50,22,2) centered at (22.8,-14.1,-13.3) with 6 peaks

with a max value of 0.0155 ALE, 1.4515588E-6 P, 4.68 Z at (22,-26,-16)

**Supplementary.** Peaks of activation in the different clusters for the sub-analysis of pleasant contrasted to unpleasant music

1: 36200 mm^3 from (-30,-38,-24) to (44,52,12) centered at (2.6,-.1,-8.3) with 23 peaks

with a max value of 0.0211 ALE, 2.2186425E-7 P, 5.05 Z at (-12,10,-6)

2: 14368 mm^3 from (-66,-42,-14) to (-30,6,22) centered at (-51.8,-17.4,4.7) with 10 peaks

with a max value of 0.022 ALE, 9.892832E-8 P, 5.2 Z at (-58,-12,6)

3: 14112 mm^3 from (30,-32,-22) to (70,16,24) centered at (49.5,-15.4,5.1) with 8 peaks

with a max value of 0.0297 ALE, 1.2150826E-10 P, 6.33 Z at (50,-20,8)

**Supplementary Table 5.** Contrasts contributing to the clusters identified in the analysis of unpleasant contrasted to pleasant music

| Cluster | Nº focis | Contrasts (studies) |
|---|---|---|
| *1* | | |
| | 1 | Kim et al., 2017. Common effect in BOLD for individual diff. in dislike of dissonant music |
| | 1 | Brattico et al., 2016. Dislike > like |
| | 1 | Menon & Levitin, 2005. Scrambled music > pleasant music |
| | 3 | Okuya et al., 2017. Fear |
| | 1 | Trost et al., 2012. Correlation with tension |
| | 1 | Koelsch et al., 2008. Irregular > regular chords |
| | 1 | Blood et al., 1999. Positive correlations with dissonance |
| | 1 | Blood et al., 1999. Negative correlations with ratings of pleasantness |
| | 1 | Blood & Zatorre, 2001. Negative correlations with pleasantness |
| | 3 | Koelsch et al., 2006. Unpleasant > pleasant |

**Supplementary Table 6.** Contrasts contributing to the clusters identified in the analysis of pleasant contrasted to unpleasant music.

| Cluster | N° Focis | Contrasts (studies) |
|---|---|---|
| *1* | | |
| | 2 | Koelsch et al., 2013. Joy > Fear |
| | 1 | Martínez-Molina et al., 2016. Pleasure ratings as a parametric effect |
| | 2 | Skouras et al., 2014. 3 Tesla. joy > fear |
| | 3 | Brattico et al., 2016. Like > Dislike |
| | 10 | Mueller et al., 2015. Correlation with pleasantness |
| | 1 | Okuya et al., 2017. Happy |
| | 1 | Trost et al., 2012. Correlation with joy, power and wonder |
| | 7 | Trost et al., 2012. Correlation with peacefulness, tenderness, nostalgia and trascendence |
| | 6 | Trost et al., 2012. Correlation with ratings of positive valence |
| | 2 | Lepping et al., 2016. Main effect of Valence. Positive > Negative |
| | 1 | Suzuki et al., 2008. Beautiful Consonance VS Ugly Dissonance |
| | 3 | Blood et al., 1999. Negative correlations with dissonance |
| | 2 | Blood et al., 1999. Positive correlations with ratings of pleasantness |
| | 4 | Blood & Zatorre, 2001. Positive correlations with chills intensity |
| | 4 | Blood & Zatorre, 2001. Positive correlations with pleasantness |
| | 1 | Flores-Gutiérrez et al., 2007. Pleasant |
| *2* | | |
| | 1 | Koelsch et al., 2013. Joy > Fear |
| | 1 | Koelsch, Skouras & Lohmann, 2018. Joy > fear |
| | 1 | Skouras et al., 2014. 3 Tesla. joy > fear |
| | 1 | Skouras et al., 2014. 1.5 Tesla. joy > fear |
| | 1 | Brattico et al., 2016. Like > Dislike |
| | 6 | Koelsch et al., 2021. Joy - Fear |
| | 3 | Mueller et al., 2015. Correlation with pleasantness |
| | 2 | Okuya et al., 2017. Happy |
| | 1 | Trost et al., 2012. Correlation with joy, power and wonder |
| | 1 | Trost et al., 2012. Correlation with ratings of positive valence |
| | 4 | Flores-Gutiérrez et al., 2007. Pleasant |
| | 1 | Koelsch et al., 2006. Pleasant > Unpleasant |
| | 1 | Altenmüller et al., 2014. Positive pieces > less positive pieces |
| *3* | | |
| | 1 | Koelsch et al., 2013. Joy > Fear |
| | 1 | Koelsch, Skouras & Lohmann, 2018. GLM results of the comparisons between emotion conditions. Joy > fear |
| | 1 | Skouras et al., 2014. 3 Tesla. joy > fear |
| | 1 | Skouras et al., 2014. 1.5 Tesla. joy > fear |
| | 1 | Brattico et al., 2016. Like > Dislike |
| | 6 | Koelsch et al., 2021. Joy - Fear |
| | 3 | Mueller et al., 2015. Correlation with pleasantness |
| | 2 | Trost et al., 2012. Correlation with joy, power and wonder |
| | 2 | Trost et al., 2012. Correlation with ratings of positive valence |
| | 2 | Lepping et al., 2016. Main effect of Valence. Positive > Negative |
| | 1 | Koelsch et al., 2006. Pleasant > Unpleasant |

In addition to our previous analyses comparing Pleasant > Unpleasant and Unpleasant > Pleasant conditions, we have conducted a contrast analysis based on these results. This analysis aims to identify which brain areas are specifically activated in the Pleasant - Unpleasant and Unpleasant - Pleasant contrasts.

Furthermore, we present the results of a conjunction analysis below, highlighting the brain regions that show overlap between these two contrasts. For all analyses, we applied a p-value threshold of 0.05, using 1000 permutations with no minimum volume threshold.

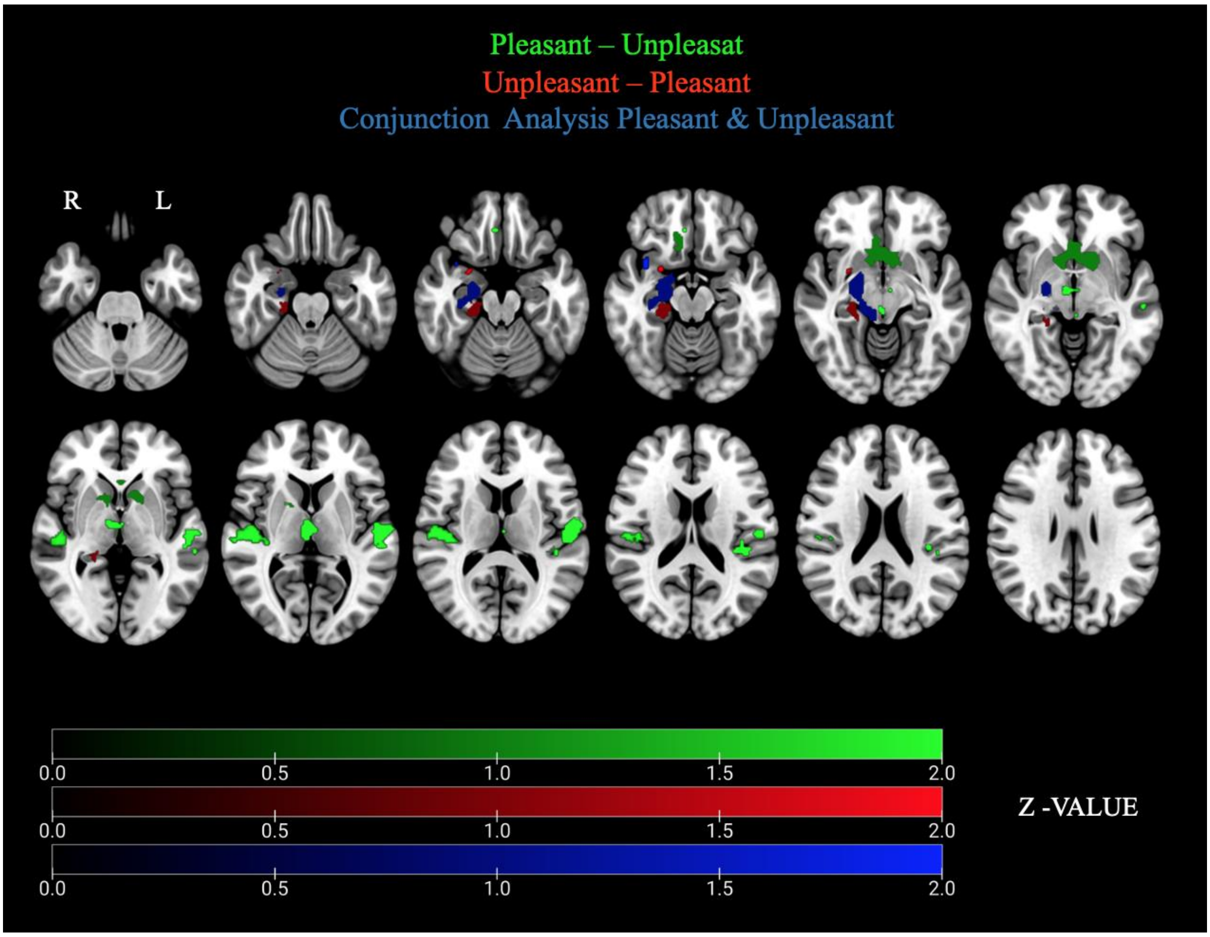


**Supplementary Figure 1**. Results from Contrast Analysis of brain regions active during listening to emotional music. In green the results of Pleasant - Unpleasant. In red the results of Unpleasant - Pleasant. In blue the conjunction analysis of Pleasant & Unpleasant. Radiological convention in coronal slices: R (right) and L (left). Gradient of the activation peaks represented according to their Z-value.

**Supplementary Table 7.** Contrast Analysis Pleasant – Unpleasant

| Cluster | Brain Region | P - value | Z - value | x | y | z | Broadmann Areas |
|---|---|---|---|---|---|---|---|
| *1* | | | | | | | |
| | L Caudate | 0.003 | 2.7 | -4.4 | 19.6 | -4.4 | - |
| | L Anterior Cingulate | 0.004 | 2.7 | -2.6 | 18.6 | -9.7 | 25 |
| | R Caudate | 0.008 | 2.4 | 12 | 10 | 2 | - |
| | L Caudate | 0.01 | 2.3 | -12 | 16 | 0 | - |
| | R Caudate | 0.016 | 2.1 | 16 | 6 | 6 | - |
| | R Caudate | 0.017 | 2.1 | 10 | 10 | -2 | - |
| | L Caudate | 0.032 | 1.9 | -4 | 9.3 | -6.7 | - |
| | R Anterior Cingulate | 0.022 | 2.0 | 4 | 28 | -10 | 24 |
| | L Putamen | 0.023 | 1.9 | -18 | 14 | -6 | - |
| | R Thalamus | 0.028 | 1.9 | 12 | 2 | 3 | - |
| | R Anterior Cingulate | 0.029 | 1.9 | 8.7 | 27.7 | -16 | 32 |
| *2* | | | | | | | |
| | R Insula | 0.007 | 2.5 | 44 | -19 | 2 | 13 |
| | R Insula | 0.009 | 2.4 | 49 | -17.5 | 8 | 13 |
| | R Insula | 0.013 | 2.2 | 44.3 | -20 | 9.7 | 13 |
| | R Claustrum | 0.009 | 2.4 | 38 | -22 | 6 | - |
| | R Insula | 0.01 | 2.3 | 48.8 | -19.4 | 16.4 | 13 |
| | R Insula | 0.014 | 2.2 | 56 | -20 | 14 | 40 |
| *3* | | | | | | | |
| | L Transverse Temporal Gyrus | 0.001 | 3.1 | -56 | -23 | 10 | 41 |
| | L Transverse Temporal Gyrus | 0.004 | 2.7 | -56 | -23.3 | 12.7 | 41 |
| | L Postcentral Gyrus | 0.006 | 2.5 | -54 | -18 | 18 | 43 |
| | L Transverse Temporal Gyrus | 0.008 | 2.4 | -56.8 | -16.4 | 12 | 41 |
| | L Transverse Temporal Gyrus | 0.008 | 2.4 | -57.5 | -13 | 10.5 | 42 |
| | L Superior Temporal Gyrus | 0.009 | 2.4 | -57.9 | -16.5 | 5.3 | 41 |
| | L Insula | 0.017 | 2.1 | -50 | -22 | 16 | 40 |
| *4* | | | | | | | |
| | L Thalamus | 0.01 | 2.3 | 0 | -18 | 2 | - |
| | L Thalamus | 0.012 | 2.3 | -2 | -14 | 2 | - |
| | R Thalamus | 0.013 | 2.2 | 5.3 | -13.3 | -2 | - |
| | R Thalamus | 0.019 | 2.1 | 6 | -18 | 4 | - |
| | L Thalamus | 0.024 | 2.0 | -2 | -10 | 4 | - |
| | L Thalamus | 0.028 | 2.0 | 0 | -12 | -6 | - |
| | R Thalamus | 0.033 | 1.8 | 4.5 | -8.5 | 5 | - |
| | L Subthalamic Nucleus | 0.04 | 1.8 | -6 | -12 | -10 | - |
| *5* | | | | | | | |
| | L Transverse Temporal Gyrus | 0.02 | 2.1 | -43.5 | -30 | 14.5 | 41 |
| | L Superior Temporal Gyrus | 0.033 | 1.8 | -36 | -32 | 15 | 41 |
| | L Insula | 0.049 | 1.7 | -44 | -34 | 20 | 13 |
| *6* | | | | | | | |
| | L Red Nucleus | 0.042 | 1.7 | 0.7 | -28.7 | -10.7 | - |
| *7* | | | | | | | |
| | R Medial Frontal Gyrus | 0.034 | 1.8 | 4 | 36 | -20 | 11 |
| *8* | | | | | | | |
| | L Superior Temporal Gyrus | 0.028 | 1.9 | -52 | -28 | 0 | 22 |

| | | | | | | | |
|---|---|---|---|---|---|---|---|
| *9* | | | | | | | |
| | L Middle Temporal Gyrus | 0.035 | 1.8 | -58 | -30 | 2 | 21 |
| *10* | | | | | | | |
| | L Precentral Gyrus | 0.046 | 1.7 | -60 | -6 | 10 | 43 |
| *11* | | | | | | | |
| | L Middle Temporal Gyrus | 0.047 | 1.7 | -60 | -34 | -2 | - |
| *12* | | | | | | | |
| | L .Middle Temporal Gyrus | 0.046 | 1.7 | -60 | -32 | 0 | - |
| *13* | | | | | | | |
| | R Putamen | 0.045 | 1.7 | 20 | 6 | 4 | - |
| *14* | | | | | | | |
| | L Insula | 0.044 | 1.7 | -36 | -28 | 22 | 13 |

**Supplementary Table 8.** Contrast Analysis Unpleasant – Pleasant

| Cluster | Brain Region | P – value | Z - value | x | y | z | Broadmann Areas |
|---|---|---|---|---|---|---|---|
| *1* | | | | | | | |
| | R Parahippocampal Gyrus | 0.005 | 2.6 | 24 | -32 | -12 | 28 |
| | R Parahippocampal Gyrus | 0.008 | 2.4 | 24 | -32.3 | -8.3 | 27 |
| | R Culmen | 0.013 | 2.2 | 20 | -28 | -24 | - |
| | R Culmen | 0.02 | 2.1 | 20 | -27 | -20 | - |
| | R Parahippocampal Gyrus | 0.021 | 2.0 | 22 | -40 | -6 | 30 |
| | R Parahippocampal Gyrus | 0.025 | 2.0 | 20 | -28 | -12 | 35 |
| | R Parahippocampal Gyrus | 0.037 | 1.8 | 20.3 | -35.5 | -1.1 | 27 |
| *2* | | | | | | | |
| | R Parahippocampal Gyrus | 0.028 | 1.9 | 26 | 2 | -22 | - |
| | R Putamen | 0.033 | 1.8 | 27.3 | 2.7 | -11.3 | - |

**Supplementary Table 9.** Contrast Analysis – Conjunction Pleasant & Unpleasant

| Cluster | Brain Region | ALE | x | y | z | Broadmann Areas |
|---|---|---|---|---|---|---|
| *1* | | | | | | |
| | R Amygdala | 0.012 | 22 | -10 | -14 | - |
| | R Hippocampus | 0.009 | 30 | -22 | -18 | - |
| | R Culmen | 0.007 | 12 | -30 | -10 | - |
| | R Substania Nigra | 0.007 | 16 | -26 | -10 | - |
| | R Culmen | 0.007 | 8 | -32 | -10 | - |
| *2* | | | | | | |
| | R Inferior Frontal Gyrus | 0.005 | 36 | 8 | -16 | 13 |
| | R Insula | 0.003 | 42 | 12 | -12 | 13 |
| *3* | | | | | | |
| | R Medial Globus Pallidus | 0.003 | 18 | 0 | -2 | - |
| *4* | | | | | | |
| | R Lateral Globus Pallidus | 0.003 | 20 | 2 | -2 | - |

In our analysis, we also aimed to explore differences in brain activation of different emotional approaches (dimensional vs. discrete) for pleasant and unpleasant emotions. Therefore, we conducted several sub-analyses that focus exclusively on the pure contrasts within each emotion model (dimensional and discrete). For the dimensional model, we have only included analyses that present pleasant > unpleasant contrasts or those where a direct correlation between neural activity and pleasantness or unpleasantness ratings is established. While for the discrete model, we have focused solely on pure contrasts between different types of emotions, such as Joy > Fear, or on direct correlations between neural activity and Joy ratings.

For these analyses, we applied a p-value threshold of 0.05, using 1000 permutations with no minimum volume threshold.

A summary of the contrasts for each emotional approach in the following table:

| | Pleasant emotions | | Unpleasant emotions | |
|---|---|---|---|---|
| | Discrete | Dimensional | Discrete | Dimensional |
| Experiments | 9 | 9 | 3 | 7 |
| Subjects | 211 | 159 | 62 | 86 |
| Focis | 79 | 69 | 21 | 46 |

Due to the N of the unpleasant studies, we do not consider that the results would have sufficient power to yield significant and consistent information. However, the results of the unpleasant contrasts are as follows:

For the neural activations during the processing of pleasant emotions induced by music:

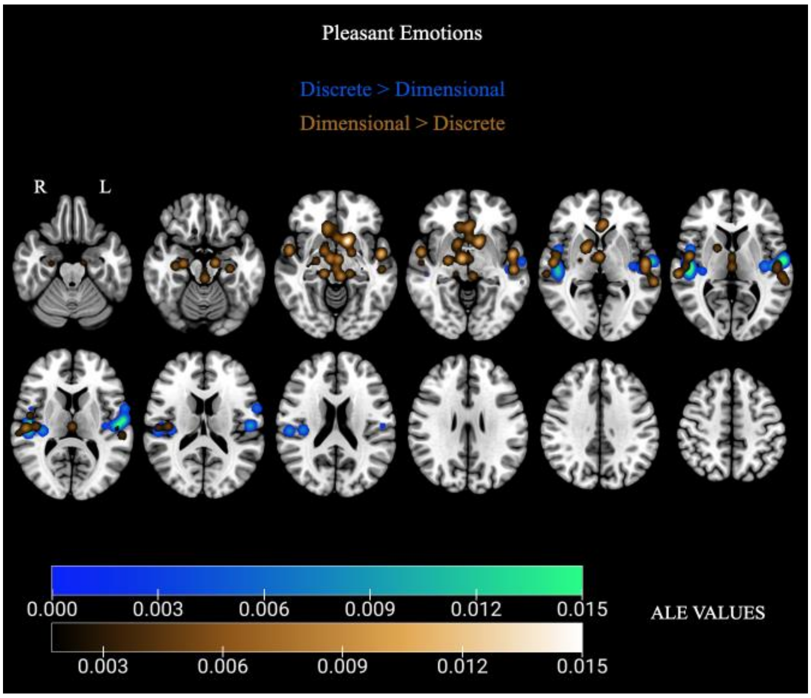


**Supplementary Figure 2.** Results from ALE meta-analysis of brain regions active for the different emotional approach for Pleasant > Unpleasant. In blue the contrast Discrete > Dimensional. In brown the contras Dimensional > Discrete.

Radiological convention in coronal slices: R (right) and L (left). Gradient of the activation peaks represented according to their Z-value.

In addition to this, we have also followed up these analyses by performing a contrast analysis between both activations for the different Discrete and Dimensional models for the contrasts of pleasant emotions induced by music. To examine whether there are statistically significant differences in the divergence and convergence between the models.

The results of this analysis can be seen below:

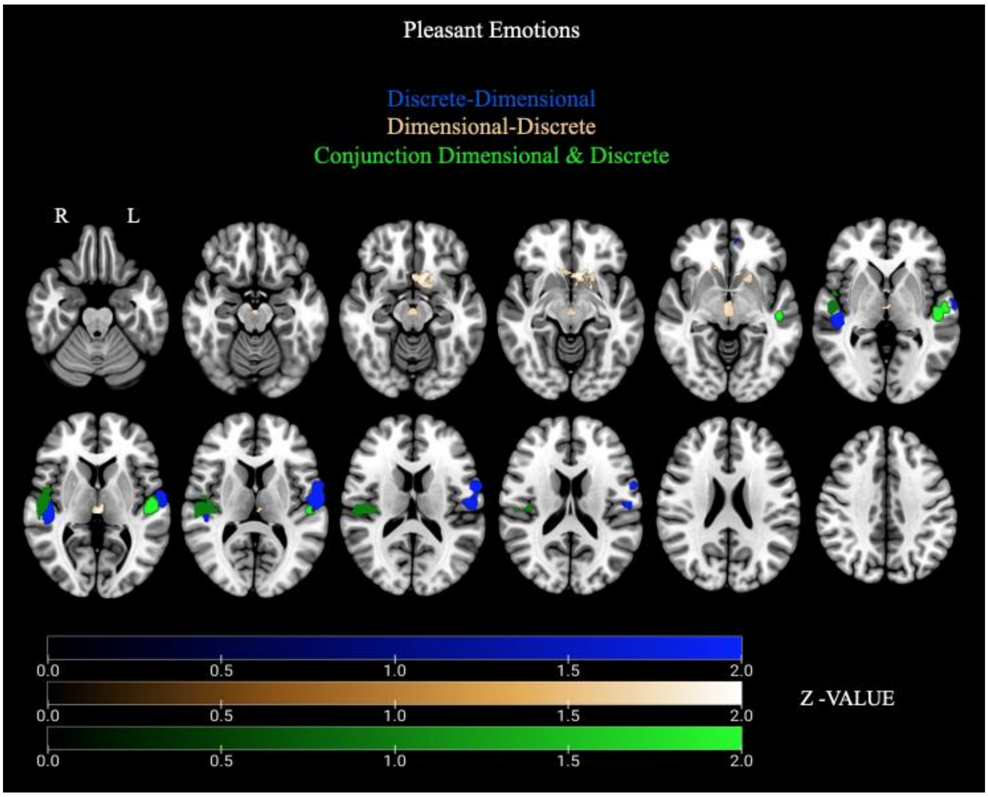


**Supplementary Figure 3.** Results from Contrast Analysis of brain regions active during listening to emotional music for the different emotional approach for Pleasant > Unpleasant. In blue the contrast Discrete - Dimensional. In soft brown the contras Dimensional - Discrete. In green the conjunction analysis between Discrete & Dimensional. Radiological convention in coronal slices: R (right) and L (left). Gradient of the activation peaks represented according to their Z-value.

Our findings revealed a consistent pattern across both the direct Dimensional and Discrete contrasts, as well as in the contrasts between them. However, this pattern should be interpreted with caution due to the limited power, with only 9 experiments in each model. The results indicate that the discrete emotion model consistently maps onto the auditory cortex, with stronger peaks in the left hemisphere, while the dimensional model shows a broader distribution across the cortex. Additionally, we identified a significant conjunction for the models in the auditory cortex of both hemispheres. Taken together, these results may provide a significant insight in the way in which different patterns of emotions are elicited in the brain through music.

**Supplementary Table 10.** For Pleasant – Discrete model of emotions

| Cluster | Brain regions | ALE | Z-value | x | y | z | Broadmann Areas |
|---|---|---|---|---|---|---|---|
| *1* | | | | | | | |
| | R Superior Temporal Gyrus | 0.025 | 6.3 | 50 | -18 | 8 | 13 |
| | R Insula | 0.009 | 3.4 | 48 | 2 | 4 | 13 |
| | R Insula | 0.009 | 3.3 | 54 | -24 | 20 | 13 |
| | R Claustrum | 0.009 | 3.2 | 34 | -24 | 10 | - |
| | R Insula | 0.008 | 3.1 | 38 | -22 | 18 | 13 |
| *2* | | | | | | | |
| | L Superior Temporal Gyrus | 0.021 | 5.5 | -60 | -12 | 6 | 22 |
| | L Superior Temporal Gyrus | 0.009 | 3.3 | -48 | -22 | -2 | - |
| | L Claustrum | 0.009 | 3.2 | -38 | -18 | 6 | - |
| | L Precentral Gyrus | 0.008 | 3.1 | -60 | 2 | 12 | 6 |

**Supplementary Table 11.** For Pleasant – Dimensional model of emotions

| Cluster | Brain region | ALE | Z-value | x | y | z | Broadmann Areas |
|---|---|---|---|---|---|---|---|
| *1* | | | | | | | |
| | L Putamen | 0.016 | 5.1 | -14 | 12 | -10 | - |
| | R Thalamus | 0.012 | 4.1 | 6 | -10 | -6 | - |
| | L Anterior Cingulate | 0.010 | 3.8 | -2 | 30 | -2 | - |
| | R Lateral Globus Pallidus | 0.009 | 3.6 | 16 | 4 | 2 | - |
| | L Thalamus | 0.009 | 3.6 | -6 | -30 | -8 | - |
| | L Mammillary Body | 0.009 | 3.6 | -2 | -10 | -10 | - |
| | R Medial Globus Pallidus | 0.009 | 3.6 | 14 | 0 | -12 | - |
| | L Thalamus | 0.009 | 3.5 | 0 | -8 | 2 | - |
| | L Lateral Globus Pallidus | 0.009 | 3.5 | -18 | -2 | -12 | - |
| | R Substania Nigra | 0.008 | 3.4 | 14 | -26 | -10 | - |
| | L Parahippocampal Gyrus | 0.008 | 3.4 | -16 | -26 | -12 | 35 |
| | L Substania Nigra | 0.008 | 3.4 | -12 | -14 | -18 | - |
| | R Caudate | 0.008 | 3.4 | 12 | 8 | -2 | - |
| | R Anterior Cingulate | 0.008 | 3.4 | 10 | 26 | -10 | 24 |
| | R Lateral Globus Pallidus | 0.008 | 3.3 | 24 | -12 | -4 | - |
| | L Red Nucleus | 0.008 | 3.3 | 0 | -22 | -12 | - |
| | R Parahippocampal Gyrus | 0.008 | 3.2 | 24 | -14 | -18 | 28 |
| | R Hippocampus | 0.007 | 3.1 | 32 | -18 | -14 | - |
| | L Hippocampus | 0.007 | 3.1 | -30 | -22 | -14 | - |
| | - | 0.007 | 2.9 | -2 | -32 | -16 | - |
| | L Anterior Cingulate | 0.007 | 2.8 | -2 | 16 | -8 | 25 |
| | L Thalamus | 0.006 | 2.8 | 0 | -20 | 8 | - |
| *2* | | | | | | | |
| | R Superior Temporal Gyrus | 0.009 | 3.7 | 54 | -2 | -6 | 22 |
| | R Superior Temporal Gyrus | 0.009 | 3.5 | 54 | -14 | 0 | 22 |
| | R Superior Temporal Gyrus | 0.008 | 3.4 | 62 | -26 | 6 | 22 |
| | R Insula | 0.008 | 3.4 | 54 | -20 | 12 | 40 |
| | R Insula | 0.008 | 3.4 | 50 | -6 | 6 | 13 |
| | R Insula | 0.007 | 2.8 | 42 | -18 | 14 | 13 |

| | | | | | | | |
|---|---|---|---|---|---|---|---|
| *3* | | | | | | | |
| | L Superior Temporal Gyrus | 0.013 | 4.4 | -52 | -4 | -8 | 22 |
| | L Superior Temporal Gyrus | 0.010 | 3.8 | -52 | -22 | -4 | - |
| | L Superior Temporal Gyrus | 0.010 | 3.8 | -52 | -14 | 0 | - |
| | L Middle Temporal Gyrus | 0.008 | 3.4 | -62 | -36 | 2 | 22 |

**Supplementary Table 12.** For Pleasant - Contrast Analysis - Discrete – Dimensional

| Cluster | Brain regions | P - value | Z - value | x | y | z | Broadmann Areas |
|---|---|---|---|---|---|---|---|
| *1* | | | | | | | |
| | L Precentral Gyrus | 0.0035 | 2.7 | -54 | -6 | 10 | 43 |
| | L Postcentral Gyrus | 0.0039 | 2.7 | -59.6 | -4.2 | 14.3 | 43 |
| | L Postcentral Gyrus | 0.004 | 2.7 | -58 | -14 | 18 | 43 |
| | L Transverse Temporal Gyrus | 0.0053 | 2.6 | -59.5 | -17 | 13.5 | 41 |
| | L Precentral Gyrus | 0.0065 | 2.5 | -52 | -12 | 12 | 13 |
| | L Superior Temporal Gyrus | 0.0117 | 2.2 | -65.3 | -7.3 | 6.7 | 22 |
| | L Superior Temporal Gyrus | 0.0132 | 2.2 | -61 | -4 | 4 | 22 |
| | L Precentral Gyrus | 0.0144 | 2.2 | -63 | 1 | 8 | 6 |
| | L Precentral Gyrus | 0.0174 | 2.1 | -65.3 | 5 | 11.7 | 6 |
| | L Superior Temporal Gyrus | 0.0261 | 1.9 | -67 | -12 | 4 | 22 |
| | L Superior Temporal Gyrus | 0.0331 | 1.8 | -68 | -12 | -2 | 21 |
| *2* | | | | | | | |
| | R Insula | 0.001 | 3.1 | 44 | -26 | -2 | 13 |
| | R Insula | 0.0029 | 2.8 | 45 | -24 | 2 | 22 |
| *3* | | | | | | | |
| | L Anterior Cingulate | 0.0431 | 1.7 | -7.4 | 51 | -4 | 10 |
| *4* | | | | | | | |
| | L Anterior Cingulate | 0.0431 | 1.7 | -4 | 48 | -3 | 32 |

**Supplementary Table 12.** For Pleasant - Contrast Analysis - Dimensional - Discrete

| Cluster | Brain regions | P - value | z - value | x | y | z | Broadmann Areas |
|---|---|---|---|---|---|---|---|
| *1* | | | | | | | |
| | L Medial Dorsal Nucleus | 0.0151 | 2.2 | -4.1 | -20.6 | 5.8 | - |
| | L Medial Dorsal Nucleus | 0.0239 | 2.0 | 1 | -18 | 4 | - |
| | L Thalamus | 0.0256 | 2.0 | 0 | -14 | 0 | - |
| | R Thalamus | 0.0266 | 1.9 | 6.7 | -15.3 | -3.3 | - |
| | L Thalamus | 0.0276 | 1.9 | -4 | -12 | 0 | - |
| | L Thalamus | 0.0331 | 1.8 | -6 | -12 | -4 | - |
| | R Thalamus | 0.035 | 1.8 | 2.3 | -15.8 | -5.7 | - |
| | R Red Nucleus | 0.0384 | 1.8 | 4 | -20 | -12 | - |
| | L Red Nucleus | 0.0388 | 1.8 | -4 | -17 | -6 | - |
| | R Mammillary Body | 0.0412 | 1.7 | 2.7 | -16.7 | -15.3 | - |
| | L Red Nucleus | 0.0442 | 1.7 | -2.5 | -18.9 | -12.9 | - |
| *2* | | | | | | | |
| | L Sub-lobar | 0.0174 | 2.1 | -6 | 10 | -14 | - |

| | | | | | | | |
|---|---|---|---|---|---|---|---|
| | L Sub-lobar | 0.0212 | 2.0 | -10 | 12 | -13 | - |
| | L Sub-lobar | 0.0194 | 2.1 | -8 | 14 | -14 | - |
| | L Putamen | 0.0205 | 2.0 | -16 | 18 | -16 | - |
| | L Anterior Cingulate | 0.0253 | 2.0 | -3.5 | 16.4 | -11.6 | 25 |
| | L Caudate | 0.0269 | 1.9 | -12.8 | 20 | -11.6 | - |
| | L Putamen | 0.0303 | 1.9 | -18 | 6 | -12 | - |
| | L Putamen | 0.0331 | 1.8 | -20 | 10 | -14 | - |
| | L Putamen | 0.045 | 1.7 | -20.4 | 17.2 | -6.8 | - |
| | L Caudate | 0.043 | 1.7 | -6 | 8 | -10 | - |
| | L Putamen | 0.0439 | 1.7 | -22 | 14 | -10 | - |
| *3* | | | | | | | |
| | R Anterior Cingulate | 0.1029 | - | 7 | 20.5 | -11.5 | 25 |
| | R Caudate | 0.043 | 1.7 | 12 | 24 | -4.2 | - |
| | L Anterior Cingulate | 0.0431 | 1.7 | 2 | 20 | -11 | 25 |
| *4* | | | | | | | |
| | R Putamen | 0.0215 | 2.0 | 22 | 8 | 4 | - |
| | R Putamen | 0.0233 | 2.0 | 22 | 0 | 4 | - |
| | R Putamen | 0.028 | 1.9 | 22 | 4 | 6 | - |
| | R Putamen | 0.0455 | 1.7 | 22 | 4 | 2 | - |
| *5* | | | | | | | |
| | L Thalamus | 0.0338 | 1.8 | -6 | -10 | 3 | - |
| *6* | | | | | | | |
| | R Lateral Globus Pallidus | 0.0362 | 1.8 | 19 | -2 | 2 | - |
| *7* | | | | | | | |
| | R Anterior Cingulate | 0.0411 | 1.7 | 8 | 22 | -18 | 32 |
| *8* | | | | | | | |
| | R Anterior Cingulate | 0.0417 | 1.7 | 8 | 26 | -12 | 24 |
| *9* | | | | | | | |
| | L Putamen | 0.0492 | 1.7 | -16 | 8 | -10 | - |
| *10* | | | | | | | |
| | R Caudate | 0.0495 | 1.7 | 12 | 24 | -10 | - |
| *11* | | | | | | | |
| | R Caudate | 0.043 | 1.7 | 16 | 26 | -6 | - |
| *12* | | | | | | | |
| | R Medial Globus Pallidus | 0.0472 | 1.7 | 16 | -2 | -2 | - |
| *13* | | | | | | | |
| | R Caudate | 0.0495 | 1.6 | 8 | 24 | -2 | - |
| *14* | | | | | | | |
| | R Medial Dorsal Nucleus | 0.049 | 1.6 | 4 | -18 | 6 | - |
| *15* | | | | | | | |
| | R Putamen | 0.027 | 1.9 | 20 | 8 | 6 | - |

**Supplementary Table 13.** Contrast Analysis for Pleasant emotions - Conjunction Discrete & Dimensional

| Cluster | Brain Region | ALE | x | y | z | Broadmann Areas |
|---|---|---|---|---|---|---|
| *1* | | | | | | |
| | R Insula | 0.008 | 54 | -20 | 12 | 40 |
| | R Superior Temporal Gyrus | 0.008 | 54 | -14 | 2 | 22 |
| | R Insula | 0.006 | 44 | -18 | 12 | 13 |
| | R Insula | 0.005 | 48 | -2 | 4 | 13 |

| | | | | | | |
|---|---|---|---|---|---|---|
| | R Insula | 0.005 | 40 | -20 | 16 | 13 |
| *2* | | | | | | |
| | L Superior Temporal Gyrus | 0.008 | -50 | -22 | -2 | - |
| | L Superior Temporal Gyrus | 0.007 | -50 | -18 | 2 | 22 |
| | L Superior Temporal Gyrus | 0.007 | -56 | -14 | 2 | 22 |
